# Current Progress in Advanced Functional Membranes for Water-Pollutant Removal: A Critical Review

**DOI:** 10.3390/membranes15100300

**Published:** 2025-10-02

**Authors:** Manseeb M. Mannaf, Md. Mahbubur Rahman, Sonkorson Talukder Sabuj, Niladri Talukder, Eon Soo Lee

**Affiliations:** 1 Department of Mechanical Engineering, Khulna University of Engineering & Technology, Khulna 9203, Bangladesh; mannaf2005103@stud.kuet.ac.bd; 2 School of Civil, Environmental, and Infrastructure Engineering, Southern Illinois University, 1230 Lincoln Dr., Carbondale, IL 62901, USA; sonkorsontalukder.sabuj@siu.edu; 3 Advanced Energy Systems and Microdevices Laboratory, Department of Mechanical and Industrial Engineering, New Jersey Institute of Technology, Newark, NJ 07102, USA; nt22@njit.edu

**Keywords:** advanced functional membranes, water purification, metal–organic frameworks (MOFs), carbon nanotubes (CNTs), electro-catalytic filtration, hybrid systems, water pollutants, dye removal, heavy metals

## Abstract

As water pollution from dyes, pharmaceuticals, heavy metals, and other emerging contaminants continues to rise at an alarming rate, ensuring access to clean and safe water has become a pressing global challenge. Conventional water treatment methods, though widely used, often fall short in effectively addressing these complex pollutants. In response, researchers have turned to Advanced Functional Membranes (AFMs) as promising alternatives, owing to their customizable structures and enhanced performance. Among the most explored AFMs are those based on metal–organic frameworks (MOFs), carbon nanotubes (CNTs), and electro–catalytic systems, each offering unique advantages such as high permeability, selective pollutant removal, and compatibility with advanced oxidation processes (AOPs). Notably, hybrid systems combining AFMs with electrochemical or photocatalytic technologies have demonstrated remarkable efficiency in laboratory settings. However, translating these successes to real-world applications remains a challenge due to issues related to cost, scalability, and long-term stability. This review explores the recent progress in AFM development, particularly MOF-based, CNT-based, and electro-Fenton (EF)-based membranes, highlighting their material aspects, pollutant filtration mechanisms, benefits, and limitations. It also offers insights into how these next-generation materials can contribute to more sustainable, practical, and economically viable water purification solutions in the near future.

## 1. Introduction

Rapid urbanization, growing industrial activities, and intensified agricultural production have resulted in the discharge of various pollutants and contaminants into freshwater bodies across the globe [1]. Key contributors to this pollution include fertilizers, pesticides, industrial effluents, and domestic sewage [2,3]. Such contamination poses serious threats to both human health and the environment [4]. In recent years, emerging pollutants (EPs), also referred to as micropollutants, have become a significant concern due to their potential risks to environmental and human health. These pollutants primarily originate from pharmaceuticals, personal care products, and hormones. They can enter water bodies either through direct discharge or by leaching into the soil [5,6]. Additionally, unsustainable plastic production, usage, and disposal practices have significantly increased the prevalence of pollutant particles known as microplastics and nanoplastics [7,8]. Microplastics (MPs) are defined as plastic particles smaller than 5 mm, while nanoplastics are even smaller typically less than 0.1 μm. These particles often carry harmful chemicals and, through interactions with other pollutants, can amplify overall toxicity [9]. To address these contaminants, various environmental remediation methods biological, chemical, and physical have been developed. Although each method varies in cost and effectiveness, they are generally more suitable for specific applications. Therefore, a combination of different approaches is often recommended for optimal pollutant removal [10].

To address the issue of microplastics and nanoplastics, specialized filtration membranes are commonly used in drinking water treatment plants (DWTP). However, over time, these very membranes can contribute to the problem by releasing micro- and nanoplastics as a result of wear and degradation. Thus, while filtration membranes are effective, they offer only a temporary solution to this challenge. For more sustainable and long-term filtration solutions, both biological and inorganic materials warrant further exploration [11]. Moreover, conventional water treatment technologies are generally effective at removing larger particles and organic matter. However, they often fall short when it comes to eliminating micropollutants [12]. In this context, advanced and eco-friendly approaches such as micro- and nano-scale material-based technologies present promising alternatives for more permanent pollutant removal [13].

A relatively cost-effective approach to removing pollutants such as dyes, heavy metals, and various chemicals from water is the use of clay and clay mineral composites. These materials are readily available and can be applied in different treatment setups. The microscale constituents of these materials adsorb contaminants based on their surface properties and swelling capacity; however, this adsorption potential is often limited. Therefore, combining clays with other substances is necessary to enhance their adsorption performance [14,15]. Activated carbon is also widely employed for pollutant adsorption in wastewater treatment, but due to its higher cost and limited practicality, its use is largely confined to large-scale industrial applications [16]. Textile industries, in particular, generate substantial volumes of wastewater laden with dyes and heavy metals. These dyes impede light penetration and increase the biological oxygen demand in water bodies. A promising and economical method for treating such dye-contaminated water is the hybrid Z-scheme system. Among the most effective solutions is the integration of multiple treatment techniques with graphene oxide, which exhibits excellent adsorption capabilities [17,18,19].

The agricultural sector is a major consumer of water. The excessive use of chemical pesticides contributes significantly to water contamination, posing serious health risks to humans. Although biodegradable and plant-based pesticides have been developed as safer alternatives, their high cost and relatively slow efficacy have limited widespread adoption [20]. In response to growing environmental concerns, industries are now mandated to treat their wastewater before discharge. This treatment typically involves three stages: primary treatment (physical processes), secondary treatment (biological processes using microorganisms), and tertiary treatment (advanced methods). However, even after undergoing all three levels, the treated water is not completely free of contaminants. While a portion of this water can be safely reused, the remainder is discharged with reduced environmental impact [21]. Primary treatment is only able to remove suspended solids and fails to act against dissolved contaminants [22]. Secondary treatment, while effective for biodegradable organics, produces large amounts of sludge as a byproduct [23]. Further handling and disposal are required for the formed sludge. Tertiary treatments include processes like advanced oxidation processes (AOPs) and conventional membrane processes. These often require high energy input and are comparatively costly to maintain overtime [24]. For these shortcomings, a need for AFMs exists to integrate selective separation, antifouling properties, and even catalytic degradation functions.

Stubborn organic pollutants from agricultural and food processing activities can be effectively degraded using the Fenton oxidation process. However, this method is limited by factors such as the requirement for acidic pH, generation of iron sludge, and high chemical consumption [25]. The integration of nanotechnology into water treatment systems has enabled the development of nanomembranes and nano-adsorbents, which offer high efficiency and superior contaminant removal capabilities. Nevertheless, concerns remain regarding their potential environmental risks with prolonged use [26]. Among these advanced materials, ultrathin graphene nanofiltration membranes (uGNMs), derived from chemically converted graphene, have demonstrated the ability to remove up to 99% of organic dyes and approximately 20–60% of salt ions. Their combination of low material demand, high performance, and cost-effectiveness makes uGNMs particularly promising for water purification applications [27]. Industries such as oil and gas, pharmaceuticals, and food processing generate substantial volumes of oily wastewater. Although treatment methods are available, the high cost hinders their practical implementation [28].

In the pursuit of more cost-effective and practical water filtration solutions, various membrane-based techniques have been developed, including ultrafiltration, reverse osmosis (RO), microfiltration, and activated carbon filtration (ACF). Among these, ACF offers a simpler and more economical alternative to the widely used RO method [29]. Membrane technologies, valued for their compactness and flexibility, have already been successfully employed in desalination plants to convert seawater into potable water [30]. In polymer-based membranes, the choice of base polymer plays a crucial role. It directly influences the optimal operating conditions for effective filtration. Incorporating nanomaterials into polymer matrices significantly enhances the membranes’ mechanical strength and contaminant rejection capabilities [31]. Additionally, to improve membrane durability, ceramic particles can be introduced. Ceramic membranes exhibit superior longevity and greater resistance to fouling compared to conventional polymer membranes [32].

Metal–organic frameworks (MOFs) are increasingly being utilized in applications such as chemical separation and sensing. MOF-based membranes have demonstrated strong potential in gas separation and are also gaining attention for liquid-phase separation processes [33]. Owing to their uniform pore sizes and tunable structures tailored to specific requirements, these membranes are highly desirable. However, challenges such as limited flexibility and the presence of structural defects can affect their performance [34]. MOFs also exhibit excellent adsorption capabilities, particularly for removing contaminants such as heavy metals and pharmaceutical residues from water [35,36,37]. While conducting an up-scaled test including 33 hollow fiber (HF) membranes, it was noted that about 73% of the membranes achieved greater than 95% rose Bengal (RB) rejection. But after incorporating an MOF layer of ZIF-93, the water permeance increased from 1.3 to 5.3 L·m^−2^·h^−1^·bar^−1^ while maintaining more than 95% dye rejection [38]. Again, using MOF-based photocatalytic membranes can also be more cost-effective as it utilizes visible light and solar energy. This reduces reliance on external energy input systems [39].

Electrochemical processes are highly effective for water disinfection, while membrane technologies excel at fluid separation. By integrating these two approaches, electroactive carbon nanotube (CNT) membranes have been developed, offering enhanced efficiency and reduced waste generation [40]. The incorporation of CNTs in varying amounts allows for precise control over membrane permeability, making them effective fillers in membrane technologies for tuning flow rates. However, due to their hydrophobic nature, CNTs tend to agglomerate, which can compromise performance. To address this limitation, functionalized CNTs have been introduced. These modified nanotubes exhibit better compatibility with membrane materials and improve filtration efficiency [41].

Despite their outstanding performance in laboratory settings, only a limited number of pilot-scale or real-water investigations have been reported to date. For MOFs, they developed an MOF–polyamide thin-film nanocomposite RO membrane that achieved more than 90% rejection of small neutral pollutants such as NDMA and boron under simulated potable reuse conditions, though still at lab scale. A notable stability demonstration was also reported for ZIF-300 membranes, which retained more than 99% Cu^2+^ rejection after 30 days in aqueous environments [42]. In the case of CNTs, progress has been closer to pilot validation. Humoud et al. demonstrated that a CNT-immobilized PTFE membrane in direct contact membrane distillation could sustain higher flux and reduced fouling when treating high-salinity produced water [43]. Almusawy et al. showed that CNT-coated sponge carriers in a pilot membrane bioreactor effectively delayed fouling and maintained stable operation with municipal wastewater [44].

Approximately 17–20% of industrial wastewater originates from dyes used in the textile industry. One effective method for degrading these harmful dyes is the photocatalytic process, which utilizes light and a semiconductor catalyst. This technique is environmentally friendly, cost-effective, and does not generate secondary waste [45,46,47]. A more recent advancement, the electro-Fenton (EF) process, has shown excellent performance in treating wastewater, particularly for the degradation of synthetic dyes like rhodamine B (Rh B). Compared to the traditional Fenton process, the EF method produces less waste and requires fewer chemicals [48]. Despite notable progress in membrane-based water treatment technologies, several challenges remain. Two major issues are membrane fouling caused by the accumulation of unwanted substances and high energy demands. Furthermore, in low-pressure systems, the operational costs often surpass the initial capital investment [49,50]. As unwanted substances accumulate on the surface of the membranes, the flux can decrease more than 50% within a few days of operation and raise the cleaning frequency and its associated costs [51,52]. Similarly, processes like seawater reverse osmosis (SWRO) consume high amounts of electricity to cause 3.90 kg CO_2_ eq/year along with 1.62 kg 1,4-Dichlorobenzene eq/year terrestrial ecotoxicity and 1.29 kg oil eq/year fossil resource scarcity [53]. By implementing strategies like surface hydrophilization, incorporation of antifouling nanomaterials, low-pressure operation with interlayers, and advanced pretreatment methods, these challenges can be somewhat mitigated as they offer improved fouling resistance and energy efficiency [54,55,56,57].

This review presents a comprehensive and critical analysis of recent advancements in the field of AFMs for water pollutant removal. Several studies reviewed membrane technologies for water purification; nonetheless, most of those studies have either concentrated on a single membrane class (e.g., MOFs or CNTs). In addition, while some studies have broadly addressed the use of nanomaterials in water treatment, they often lack detailed perspectives. There remains a critical need for a comprehensive review on AFMs that connects molecular-level structural details with their functional performance a gap that has yet to be filled. Furthermore, the existing literature lacks clear comparative analyses of different types of AFMs to highlight their respective strengths and weaknesses. Information on their long-term performance under real water conditions remains limited, and studies addressing cost analysis, scalability, and the integration of AFMs into hybrid systems are still scarce.

This study presents a comparative and critical evaluation of three emerging AFM-based technologies, namely MOF-based membranes, CNT-based membranes, and electro-Fenton/electro–catalytic hybrid membranes. Particular emphasis is placed on the innovative aspects of material design, synthesis strategies, functional mechanisms, and their impact on performance. By critically evaluating the structural and operational features of these membranes, this review underscores their potential in addressing a wide range of emerging pollutants, including heavy metals, pharmaceutical residues, per- and polyfluoroalkyl substances (PFAS), microplastics (MPs), etc. This work aims to offer guidance for future research and practical implementation of AFMs in sustainable water treatment technologies.

## 2. General Concept of Advanced Functional Membranes

**Figure 1:**
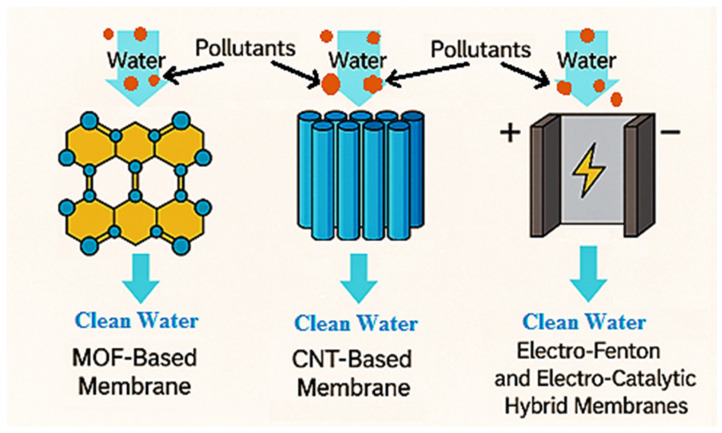
Schematic representation of Advanced Functional Membranes.

Advanced Functional Membranes are a class of engineered next-generation membranes with unique physicochemical features that can be effectively employed for generalized and specified activities for wastewater treatment. AFMs generally integrate nanostructured materials or catalytic components. These additions provide specific functional properties such as adsorption, photocatalysis, electrocatalysis, or charge-based separation in the matrix of the membrane. Among the AFMs, this review is specifically focused on CNT, MOFs, and electro-Fenton integrated membranes, as presented in Figure 1 in a general context. CNT membranes have also gained significant attention in recent days. CNTs in the form of buckypapers and composite materials have a variety of uses when it comes to filtration. On another side, MOFs are a class of materials made up of metal ions and organic molecules that create porous structures, allowing them to have a large and flexible surface area and customizable properties [58,59]. MOFs like UiO-66 and UiO-66-NH_2_ are integrated with polymers like polyethersulfone (PES) to make mixed matrix membranes (MMMs) more resistant to fouling. This not only makes the membranes resistant to fouling but also improves the flow of water [60,61]. In addition to these membranes, electrochemically active membranes, particularly those based on electro-Fenton (EF) and electro–catalytic mechanisms, have emerged as powerful tools for the removal and degradation of organic pollutants in water over a wide range of pH [62].

**Figure 2:**
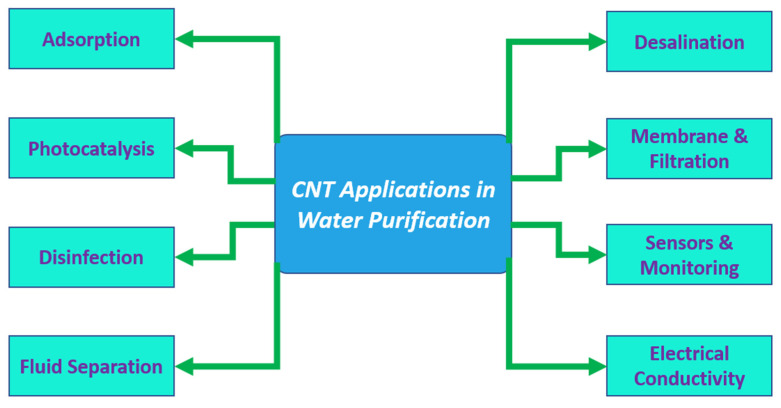
Applications of carbon nanotubes (CNTs) in water purification.

CNTs allow air and water to pass through while effectively separating small molecules based on size. With it also being comparatively easier to produce, it is an excellent option for both small- and large-scale filtration purposes [63,64,65]. Depending on the specific criteria in which the CNT membranes are to be used, the CNTs can be placed either horizontally or vertically. This arrangement allows for proper flow and minimal fouling [66]. For the removal of water from kerosene, a new type of filter called a carbon nanotube immobilized membrane (CNIM) is now being tested. The CNTs, being hydrophobic, reject the water to pass at a rejection rate of 99% while allowing kerosene to pass. This separates the kerosene from the water pretty successfully, leaving filtered water behind [67]. Different test results show that the porosity and thermal stability of CNT-mixed PES membranes increase with the addition of CNTs. Among the test specimens with 2.5%, 5%, 7.5%, and 10% CNT, filtration tests show that the membrane with 5% CNT performed the best in achieving a higher flow rate while effectively removing contaminants [68]. Sustained performance and reusability make CNTs an excellent candidate for practical water treatment applications, as presented in Figure 2.

A certain type of MOF, such as zeolitic imidazolate frameworks (ZIFs), has gained considerable attention for water filtration technologies because of its high thermal and chemical stability. ZIF-8, in particular, has shown excellent results for hydrocarbon separation, while ZIF-7 is being used for selective lithium extraction, capitalizing on its ion exchange capacity [69,70]. Membranes coated with ZIF-8-PDA can also be used for lithium-ion extraction from sodium (Na) and potassium (K) [71,72]. Among the various MOF-based materials, the best separation and filtration performances were achieved by membranes adding MOFs into polycrystalline structures or 2D nanosheet membranes [73].

Electro-catalytic membrane reactors (ECMRs) are a new approach to water treatment that utilizes porous electrodes to enhance the breakdown of persistent organic pollutants (POPs) [74]. Similarly to ECMRs, photocatalytic membrane reactors (PMRs), while working on the same principle, utilize light to break down harmful pollutants. The photocatalysts in this case (TiO_2_ or ZnO) can either be mixed in the solution or attached to the membrane itself [75,76]. A common antibiotic called sulfadiazine (SDZ) is actually a very harmful micropollutant. To remove it from wastewater, photo-Fenton ceramic membranes are required. Using a combination of UV light and a catalyst known as goethite, the removal rate of SDZ can be significantly improved. And an addition of hydrogen peroxide on top of all these boosts the removal capacity up to 99% CVD (electrocatalytic) properties into one membrane. Photo–electro–catalytic membranes developed from stainless steel with a coating of semiconductor materials. These membranes are greatly effective in fighting and filtering the persistent organic pollutants (POPs) [77].

Perfluorooctane sulfonic acid (PFOS) and perfluorooctanoic acid (PFOA) are two very harmful chemicals that can cause cancer and are very difficult to separate from water. Using specially designed aluminum oxide filters, over 90% of this PFOS and PFOA can be removed. By the addition of hydrophilic components, the pressure is reduced during filtration, and it helps to ensure a high contaminant removal rate [78,79]. Stavudine, an antiretroviral drug found in wastewater, which is not easily filtered out by traditional treatment, can now be broken down using an electrochemical process with an anode made of Ti/SnO_2_-Sb. The degradation of stavudine is seen to follow a predictable pattern with a rate constant of 0.24 min^−1^ and a half-life of 2.9 min at a certain current density. However, the presence of ions like nitrates and chlorides can inhibit the degradation process, causing it to occur at a slower rate, which leads to the formation of some byproducts that turn out to be more toxic than the original contaminant, stavudine [80].

## 3. Metal–Organic Framework (MOF)-Based Membranes

Metal–organic frameworks (MOFs) are an emerging class of crystalline materials constructed by coordination of metal ions or metal-containing clusters with organic ligands. Since their emergence in the late 1990s, MOFs have attracted significant attention owing to their exceptionally high surface areas, frequently surpassing 1000 m^2^/g [81,82]. Highly porous MOFs are also known for their stable three-dimensional frameworks, tunable pore structures, and adjustable surface functionality [83]. These features, engineered through different synthesis methods, make MOFs exceptionally attractive for applications ranging from gas storage and catalysis to sensing and water purification in comparison to existing techniques [81,84]. Particularly, in the field of water purification and membrane science, conventional polymeric membranes are often limited by low selectivity, poor tenability, and trade-offs between permeability and rejection. Ceramic membranes, on the other hand, provide good chemical and thermal stability but are costly and largely inert [29]. Against this backdrop, MOFs bring new possibilities by integrating adsorption, separation, and catalysis within a single material platform [85]. Numerous contaminants, including forever chemicals, have been effectively removed through pollutant-specific mechanisms. An in-depth discussion of these pathways and the associated challenges is provided in this section.

### 3.1. MOF-Based Membranes in Water Purification

Traditional membranes are generally based on polymers or inorganic oxides. Typically, these membranes face drawbacks such as low selectivity, poor tunability, and tradeoffs between permeability and rejection [29]. On the other hand, MOF-based membranes offer molecular-level precision, enabling control over high selectivity and high permeability simultaneously. MOFs can be tailored with pore apertures ranging from a few angstroms to several nanometers. This allows precise size-based separation of contaminants, including heavy metals, dyes, and emerging micropollutants. Additionally, MOFs can incorporate chemically reactive sites either during synthesis or via post-synthetic modification. Functional groups such as –NH_2_, –COOH, –OH, and even catalytic metal centers can be introduced to facilitate specific interactions with target pollutants in wastewater [86]. Moreover, in some MOFs, integration of transition metals such as Fe, Cu, or Ti exhibits photocatalytic properties such as photocatalysis or Fenton-like redox activity. These chemically active MOFs can degrade pollutants while simultaneously offering physical removal thanks to their dual functionality [39]. Another advantage of MOFs is their structural responsiveness. Some specific MOFs show ‘breathing’ behavior, where pore structures expand or contract depending on the adsorbed molecules. This offers potential for adaptive separation of pollutants from wastewater [87].

Initially, MOFs were studied as powdered absorbents by dispersing MOF particles into polluted water, allowing pollutant removal through adsorption and degradation. However, this approach has some limitations, such as poor reusability, slow kinetics, and insufficient recovery of MOFs after treatment. Hence, researchers started to incorporate MOFs directly into membranes to overcome these hurdles [88]. Several techniques, such as coating, grafting, and polymer embedding, were used to integrate MOFs within membranes in a controlled manner, expanding their role from passive sorbent to active membrane components capable of selective separation and photocatalytic degradation [89]. This shift established MOFs as an integral element in modern membrane-based techniques for water purification.

Among all the MOFs developed so far, a few types, including ZIF-8, UiU-66, and MIL-series, are being extensively studied for membrane-based water treatment due to their potential for functionalization and chemical and thermal stability in aqueous systems. The surface properties of MOFs mainly depend on the synthesis method and the defects present in the MOF. Different structural and chemical characteristics of ZIF-8, UiO-66, and MIL-101(Cr) are presented in Table 1.

### 3.2. Synthesis Strategies of MOF-Based Membranes

Generally, three strategies have been widely employed to incorporate MOFs into membranes: in situ growth, layer-by-layer (LBL) assembly, and mixed matrix membrane (MMM) fabrication. In situ methods promote strong MOF-substrate integration, LBL offers precise control over structure, while MMM’s approach can blend MOF functionality with polymer flexibility. While each method presents unique advantages, the synthesis approaches must address the issues related to scalability, fouling resistance, and long-term operational stability, etc. [98]. Some relatively new techniques, such as electrospinning and 3D printing, are also offering promising solutions to MOF-based membrane technologies [99].

**Figure 3:**
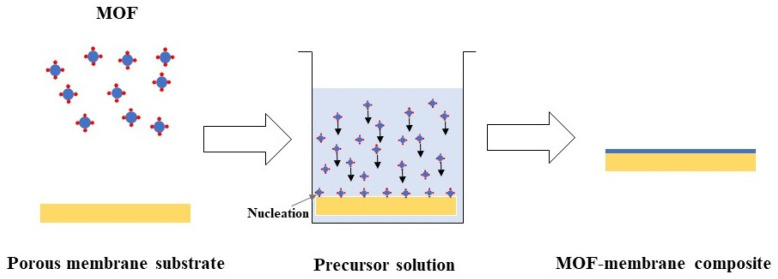
Schematic of in situ growth approach to prepare MOF-based membrane.

In the in situ growth approach, as depicted in Figure 3, MOF crystals are formed directly on or within a porous substrate by immersing the support in a precursor solution containing metal ions and organic linkers. This can be performed via solvothermal, hydrothermal, or room temperature aqueous conditions, depending on the solubility and reactivity of the precursors and the thermal stability of the substrate [100]. Formation of minimal defect continuous MOF film is the key advantage of this approach. This formation generally follows a bottom-up nucleation and crystal growth mechanism. The formation of MOF crystals is influenced by many factors, including precursor concentration, pH, surface modifiers, presence of nucleation promoters, and synthesis time [101].

Several MOFs, including ZIF-8, UiO-66, and HKST-1, have been successfully grown on different supports such as polymeric membranes, alumina, stainless steel meshes, and graphene oxide films. For instance, in situ growth of ZIF-8 on polyacrylonitrile support resulted in membranes with excellent nanofiltration capability due to ZIF-8’s small pore size and hydrophobicity [102]. Similarly, UiO-66 grown directly onto ceramic supports successfully removed heavy metals and pharmaceuticals from an aqueous matrix [103]. However, the in situ growth technique has some cons, such as low nucleation efficiency, large chemical requirement, uniform coating, and, most importantly, difficulty in controlling the thickness and crystal orientation of the film [100]. Numerous research initiatives are trying to address these issues, focusing on membrane-based wastewater treatment.

**Figure 4:**
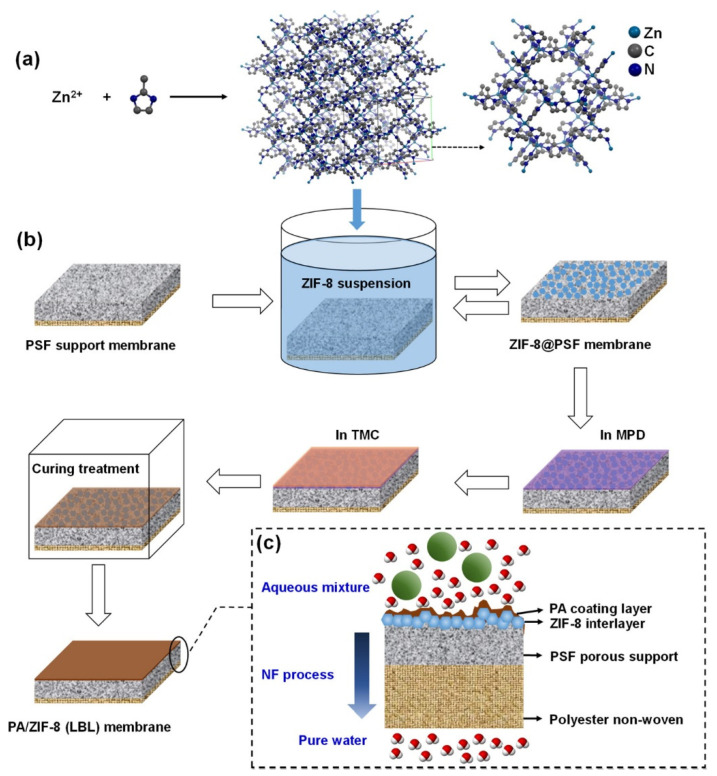
Schematic of LBL membrane fabrication (ZIF-8 in blue, MPD in purple, TMC in orange, and PA in brown): ZIF-8 synthesis (**a**), LBL fabrication procedures (**b**), and cross-sectional structure of the PA/ZIF-8 (LBL) membrane (**c**). Reprinted with permission from [105]. Copyright 2015 American Chemical Society.

The LBL assembly offers molecular-level control over thickness, crystallinity, and orientation by sequential deposition of metal ions and organic linkers on the substrate. Each cycle of deposition with intermediate rinsing deposits a thin layer of MOF material [104]. Polyamide/ZIF-8 nanocomposite prepared using the LBL approach illustrated in Figure 4 achieved a 99.8% dye rejection rate [105]. Gao et al. deposited UiO-66-NH_2_ MOF coatings on a polymer support via an LBL liquid-phase epitaxy method. This ultra-thin membrane displayed excellent oil-water separation performance [106]. However, the slow and labor-intensive LBL approach has scalability issues for ubiquitous usage of this method for water pollutant removal.

**Figure 5:**
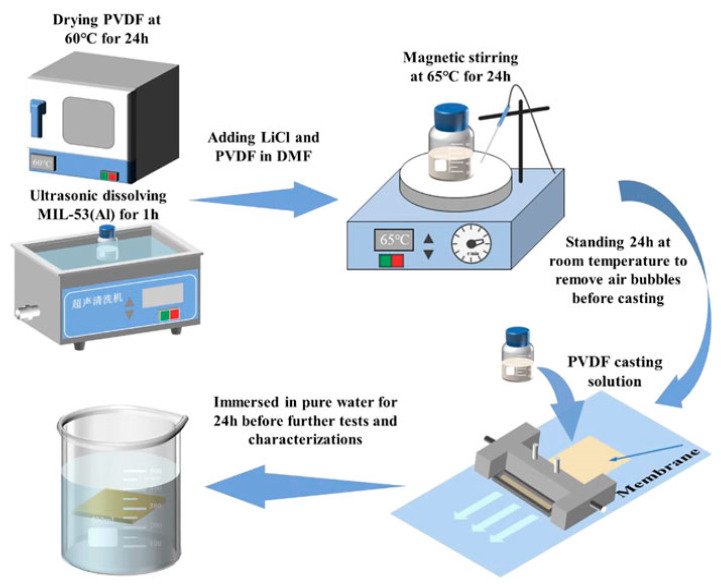
Synthesis of PVDF/MIL-53 (Al) membrane via MMM approach [107].

Compared to LBL, MMM is the most industry-relevant and scalable method to integrate MOFs into membranes for water purification. In MMM, MOF particles are dispersed into polymer solution, followed by membrane casting (via phase inversion, spin coating, or solvent evaporation) or extrusion into hollow fiber or flat sheets. ZIF-8, UiO-66, and MIL MOFs are commonly used in MMM due to their high surface area and chemical stability [98]. As shown in Figure 5, MIL-53 (Al) particles dispersed with polyvinylidene fluoride (PVDF) in dimethylformamide (DMF), followed by membrane casting. This improved the hydrophilicity, and the ultrafiltration membrane showed excellent antifouling performance in water treatment [107]. In a similar approach, a ZIF-8-coated polyvinylidene fluoride membrane was synthesized using the MMM approach, and it could treat wastewater released from the pulp and paper industry with significant efficacy [108]. Nonetheless, a key challenge in the MMM approach lies in achieving strong interfacial compatibility between the MOF fillers and the polymer matrix. Inadequate dispersion or agglomeration of MOF particles may lead to the formation of nonselective voids or interfacial defects [109]. To mitigate these challenges, several strategies have been employed, such as functionalizing the MOF surface to improve compatibility, introducing compatibilizing agents to promote uniform dispersion, and applying post-fabrication thermal annealing to strengthen interfacial adhesion [110].

### 3.3. Pollutant Removal Mechanisms

MOF integrated membranes are found to be effective in the removal of a diverse class of water pollutants, including heavy metals, dyes, antibiotics, and PFAS. Their modular framework allows surface functionalization, tailored pore sizes, and integration of catalytic metallic centers, all of which contribute to pollutant-specific removal pathways. However, most studies have been performed in simplified aqueous systems; hence, the selectivity limits for different pollutants using MOF-based membranes can be incorrect in realistic water matrices containing competing ions, natural organic matter, and variable pH conditions. Further systematic testing under practical environments is therefore required to better understand selectivity and underlying mechanisms. The following sections discuss the mechanisms of these pathways for key pollutants.

#### 3.3.1. Heavy Metals

**Figure 6:**
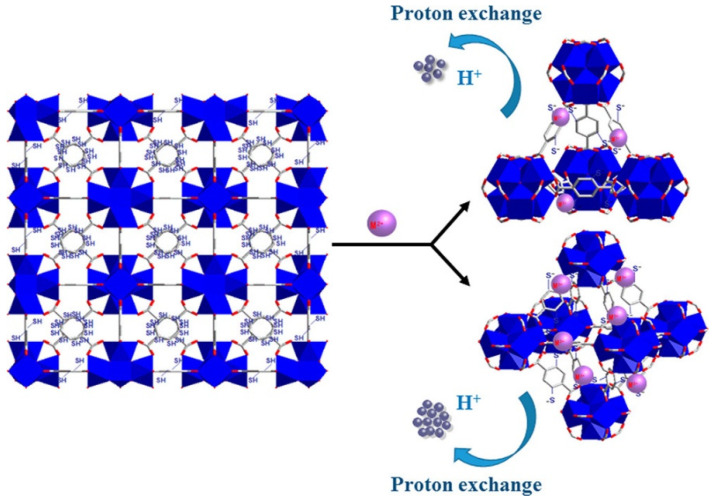
‘Proton exchange’ facilitating Hg^2+^ capture using thiol groups. Reprinted with permission from [112]. Copyright 2018 American Chemical Society.

Heavy metal ions such as lead (Pb^2+^), chromium (Cr^6+^), mercury (Hg^2+^), arsenic (As^3+^ and As^5+^), and cadmium (Cd^2+^) are persistent, toxic, and non-biodegradable inorganic contaminants present in water sources. Trace-level presence of heavy metals can cause serious health issues due to their tendency to bioaccumulation. MOF-based membranes can be employed to remove these heavy metallic substances primarily through chelation and electrostatic interaction [111]. MOFs with carboxyl, amino, or thiol functional groups can bind metal ions through coordinate bonding. As shown in Figure 6, MOFs containing thiol-modified frameworks can bind soft metal cations like Hg^2+^ via sulfur affinity [112]. Similarly, UiO-66-NH_2_ has demonstrated strong binding affinity toward Pb^2+^ and Cr^6+^ through nitrogen lone pair coordination and electrostatic attraction [113].

In addition to surface functional groups, MOF membranes with negatively charged surfaces can selectively attract cationic heavy metals while repelling competing anions. This pathway highly depends on solution pH, metal speciation, and membrane surface charge. As an example, UiO-66 integrated polysulfone ultrafiltration membrane was found to be highly effective in selective removal of Sr^2+^, Pb^2+^, Cd^2+^, and Cr^6+^ [114]. Although MOF-integrated membranes could selectively remove heavy metals via chelation and electrostatic attraction, most of these studies were conducted in standard aqueous conditions. It is now essential to focus on environments with competing ions and the presence of natural organic matter to replicate practical cases. In addition, many MOFs exhibit pH-sensitive behavior, with acidic or oxidative conditions that could possibly degrade linkers or promote metal leaching, a critical concern when targeting redox-active metals like Cr^6+^.

#### 3.3.2. Organic Dye Removal

Organic dyes like methylene blue (MB), rhodamine B (RhB), methyl orange (MO), crystal violet (CV), and Congo red (CR) are resistant to biodegradation and hazardous even at low concentrations. MOF-based membranes showed promise in removing dyes through several pathways, including size exclusion, adsorption via electrostatic interaction, π–π interactions, and catalytic degradation. Size exclusion can be effective when the molecular dimensions of the dye are greater than the pore aperture of the MOF. For instance, ZIF-8 with a narrow aperture embedded in a polysulfone membrane rejects organic dyes with a higher molecular size than ~3.5 Å, which covers most of those molecules [115]. On the other hand, larger-pore MOFs like MIL-101(Cr) (pore aperture 12–16 Å) can trap dye molecules within their mesoporous cages and restrict their exit. This selective entrapment offers effective removal of organic dyes from wastewater [97].

**Figure 7:**
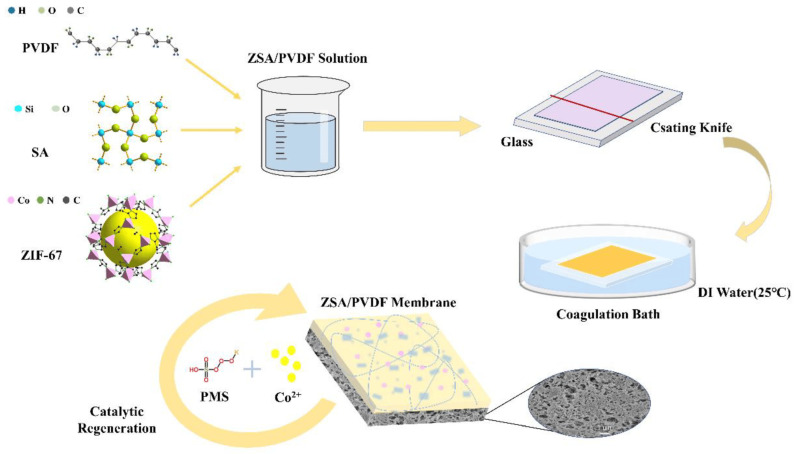
ZIF-67/SA@PVDF ultrafiltration membrane with simultaneous adsorption and catalytic oxidation for dyes [117].

In addition, some membranes exhibit adsorptive removal of organic dyes when modified with MOFs, particularly ZIFs. Aromatic MOFs provide π–π stacking interactions with dye molecules, and charged or functional groups on MOFs show electrostatic attraction [116]. For instance, Zhu et al. incorporated ZIF-67 and porous silicon aerogel into polyvinylidene fluoride (PVDF) membrane, as seen in Figure 7. This modified membrane removed more than 99% cationic MB through π–π and n–π interactions between MB and the MOF. On the other hand, a lower removal rate was observed for anionic Acid orange 7 dye. This implies that electrostatic attraction between the negative membrane surface and cationic MB enhanced the adsorption [117]. Another study conducted by Li et al. also suggests a similar pathway for RhB removal using a UiO-66-SO_3_H-modified nylon 6 membrane [118,119]. Additionally, some MOF-based membranes demonstrate photocatalytic degradation of organic dyes. For example, ZIF-8 modified with Ag nanoparticles, supported on a stainless-steel mesh, degraded RhB under visible irradiation [120].

Though dye removal through various mechanisms has been reported already, some important questions need to be answered to fully understand the removal potential. Firstly, while size exclusion by ZIF-8 is frequently cited, actual dye rejection depends not only on molecular size but also on hydration shell, molecular flexibility, and pore accessibility, which are often overlooked. Likewise, photocatalytic membranes’ stability, radical generation efficiency, and reusability need to be studied under continuous flow and real effluent conditions.

#### 3.3.3. Antibiotics and Pharmaceuticals

Antibiotics such as tetracycline (TC), ciprofloxacin (CIP), and sulfamethoxazole (SMX) and various pharmaceutical compounds like salicylic acid (SA) and diclofenac (DCF) are commonly found in surface water, groundwater, and wastewater. These contaminants cause serious harm to the environmental ecosystem via antibiotic resistance. Conventional treatment processes often fail to completely remove these hazardous substances.

**Figure 8:**
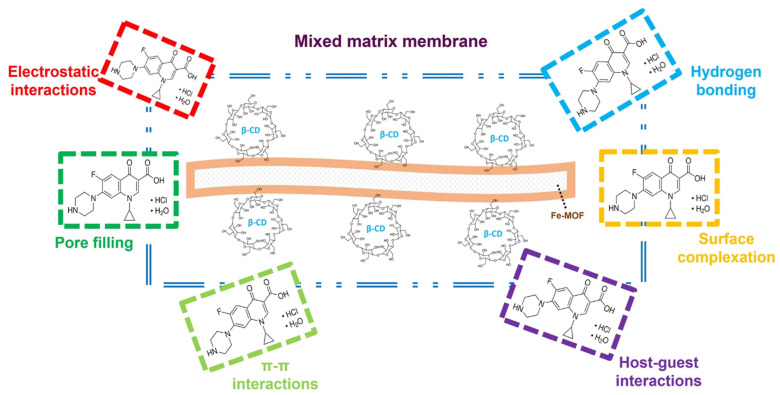
Adsorption mechanism of CIP using Fe-MIL-88B/PVDF MMM [121].

MOF-based membranes offer an effective solution for removing these pollutants through various mechanisms, including adsorption, molecular sieving, and catalytic degradation. This type of membrane has demonstrated excellent adsorption of pharmaceuticals, which it attributed to MOF/pharmaceutical interactions, including electrostatic, hydrogen bonding, π–π stacking, and the high porosity of MOF-based membranes. β-Cyclodextrin-functionalized Fe-MIL-88B/PVDF MMM removed 87.6% of CIP following monolayer adsorption, as shown in Figure 8. Amino groups present in MOF interact with the carboxyl group of CIP to adsorb the pollutant via electrostatic interaction [121]. UiO-66(Hf)-NH_2_ metal–organic framework grown on electrospun polymer nanofibers completely removed SA from a 50 mg/L solution via hydrogen bonding and π–π stacking. The MOFs provide Lewis-acidic metal sites that can coordinate the SA’s carboxylic groups, while the amino and hydroxyl groups form hydrogen bonds. Additionally, the aromaticity pores allow π–π interactions with the SA benzene ring [122]. Similarly, adsorptive removal of TC was achieved by ZIF-8-grown regenerated cellulose nanofibers through a synergetic action of coordination, hydrogen bonding, π–π stacking, and electrostatic interactions [123].

In catalytic MOFs, especially those containing transition metals like Fe or Ti, the removal mechanism also involves photocatalytic or Fenton-like degradation. These reactions can break down pharmaceuticals into shorter compounds. For example, MIL-53 (Fe)-embedded PVDF MMM removed 99% TC from water via photocatalysis under visible light irradiation. Holes and OH radicals are found to be the dominant species that drove the TC decay following pseudo-first-order kinetics [124]. Additionally, a study reports that the ZIF-8 composite membrane can separate 99% of TC through molecular sieving [125].

Various mechanisms have already been reported for the removal of antibiotics using MOF-embedded membrane, yet many key areas remain unexplored. While adsorption driven by electrostatics, hydrogen bonding, and π–π interactions is frequently reported, these interactions are highly dependent on solution conditions such as pH, ionic strength, and the presence of co-contaminants. For instance, the strength of MOF–drug interactions can shift drastically in wastewater containing surfactants or competing solutes. The effect of these solution conditions needs to be explored to fully understand the adsorptive removal of antibiotics using MOF-based membranes. For catalytic removal, though impressive degradation efficiency has already been reported in the literature, the durability of reactive MOFs under prolonged exposure has not been evaluated yet. Additionally, the fate of degradation byproducts, which in some cases could be more hazardous than the mother compounds, requires in-depth investigations.

Beyond membrane durability, an equally critical issue is the toxicity of degradation byproducts generated during antibiotic removal. While MOF-based catalytic membranes can achieve nearly complete removal of parent compounds, the intermediate products formed during photocatalytic or Fenton-like reactions with MOF may retain or even exhibit greater toxicity than the original pollutant. For example, MOF-based photocatalysis of lincomycin showed increased toxicity at early stages due to the formation of more harmful intermediate byproducts [126]. Similarly, Han et al. reported that photo-Fenton degradation of oxytetracycline generated a transformation product with higher predicted toxicity, as indicated by QSAR analysis [127]. Despite these potential risks, few studies have systematically identified transformation products or evaluated their ecotoxicity. Therefore, future research should couple degradation efficiency tests with advanced chemical analysis and bioassays to ensure that MOF-based catalytic membranes deliver both effective removal of antibiotics and pharmaceuticals and environmentally safe treatment outcomes.

#### 3.3.4. Per- and Polyfluoroalkyl Substances

**Figure 9:**
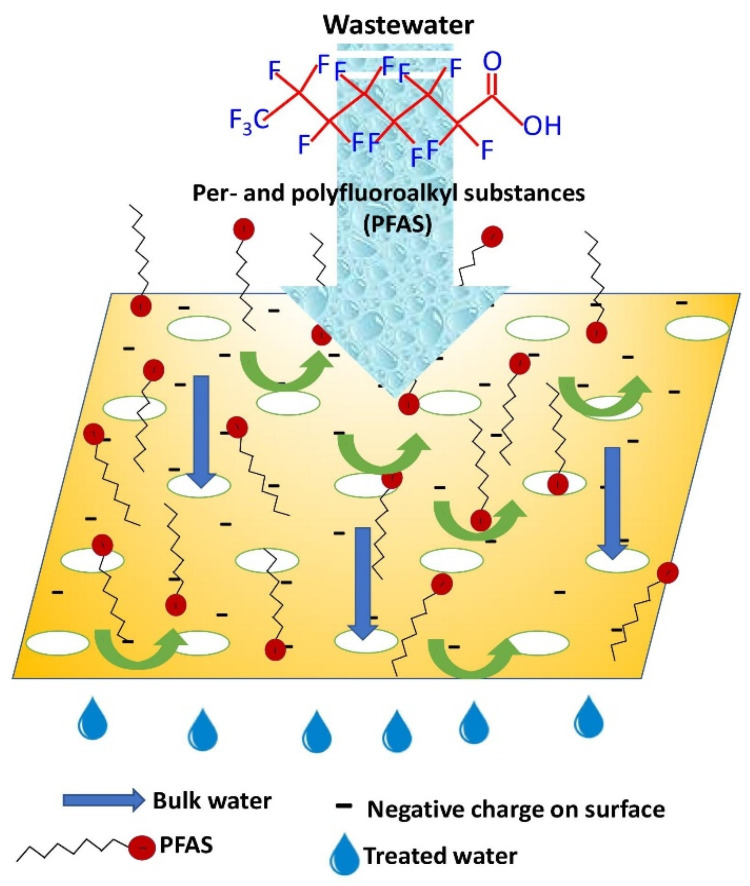
PFOA removal mechanism using MIL-100(Fe) embedded MMM [130].

Per- and polyfluoroalkyl substances, commonly known as PFAS, are a group of persistent pollutants that are classified as forever chemicals due to the presence of strong C-F bonds. These substances are linked to causing cancer, are non-biodegradable, and cannot be treated by traditional water treatment techniques [128]. MOF-based membrane technology offers us an emerging solution to remove this PFAS from water. To begin with, a polyamide-modified novel MOF membrane effectively removed 84% of shorter-chain PFAS and 95% of longer-chain PFAS from water. Electrostatic repulsion, size exclusion, and adsorption via hydrogen bonding between the PFAS carboxyl/sulfonate groups and the hydroxyl or phosphate groups on the MOF surface contribute to this filtration [129]. MIL-100(Fe)-embedded polydomaine-modified MXene-based MMM successfully removed PFAS from actual wastewater. The study reveals that size-based exclusion occurred as the PFAS molecules’ size increased by forming micelles, as shown in Figure 9. Additionally, adsorption also occurred via hydrogen bond formation between the carboxyl group and PFOA. Electrostatic repulsion between the negative membrane surface and PFAS molecules also contributed to the removal process [130].

Another PVDF membrane combined with CuBTC was able to remove 79.64% perfluoro heptanoic acid through hydrophobic interaction [131]. A different study reported that aluminum fumarate MOF contributes to the PFOA adsorption capacity of polytetrafluoroethylene (PFTE) membrane [132]. ZIF-L-loaded polyethyleneimine membrane also successfully removed shorter and longer chain PFAS from contaminated water. Density functional theory (DFT) calculations reveal that MOF’s electrostatic interaction, hydrogen bonding, and van der Waals interactions have contributed to the PFAS removal [133].

Multiple mechanisms for PFAS removal using MOF-incorporated membranes have been cited, but in-depth studies about these pathways and their specific contributions have not been explored yet, except for a few studies [129]. Fundamental understanding of interactions of PFAS with MOF surface and pore structure is required to design MOF-based membranes for PFAS removal. In addition, specific co-contaminant interactions, including metal ions, surfactants, and other organic pollutants, are yet to be explored. In addition, regeneration studies (e.g., solvent cleaning and backwashing) of membranes after repeated PFAS loading are also to be studied.

### 3.4. Current Challenges

**Figure 10:**
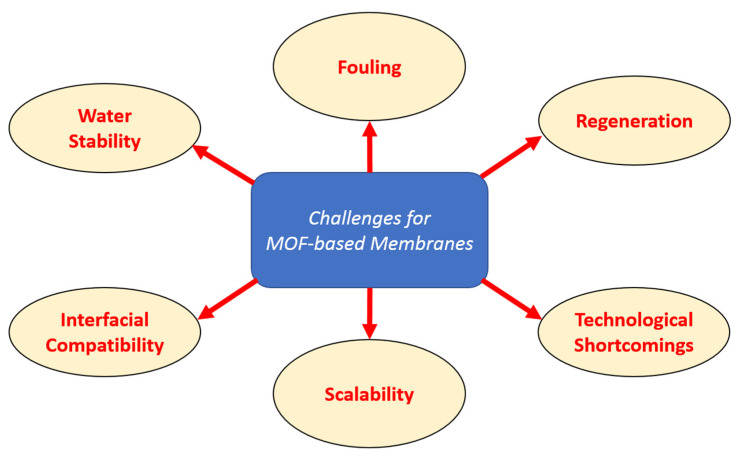
Current challenges in the field of MOF-based membrane for water purification.

Thanks to their exceptional ability to simultaneously separate, adsorb, and catalytically degrade pollutants, MOF-based membranes offer an edge over conventional membrane technologies. Despite these advantages, this emerging technology has some technical, economic, and operational challenges, as shown in Figure 10, which need to be addressed before enabling long-term scalable applications. The most pressing limitations are water stability, membrane fabrication and cost, interfacial compatibility, fouling resistance, and regeneration.

Water stability is one of the most fundamental limitations of MOF-incorporated membranes. In real wastewater, the presence of multiple competing ions, surfactants, and organic solvents can make this issue more critical. Many MOFs, particularly Zn-based ones, often exhibit structural degradation in water. This can collapse the membrane system and lead to leaching of metal ions to the environment. For instance, alumina-supported ZIF-8-based membranes have been found to leach Zn^2+^ and gradually degrade in water at room temperature. This structural collapse was due to hydrolysis of ZIF-8 [92]. Another study supports this instability, as crystals present in ZIF-L coating on stainless steel mesh were found to decompose regardless of the pH of water [102].

Another key engineering challenge in MOF-embedded membranes, particularly in MMMs, is compatibility issues between the MOF particles and the polymer matrix. Poor dispersion or mismatched surface energies can result in interfacial voids or nonselective defects, reducing membrane selectivity and mechanical integrity [134]. To mitigate this, several techniques, including surface functionalization of MOFs, polymer blending, and interfacial annealing, are employed [110,135,136,137,138]. However, these steps add complexity to fabrication and may not be scalable for industrial use. In addition, MOF particle aggregation during membrane casting or drying can lead to performance heterogeneity [34]. To further illustrate the performance variability of different AFMs, Table 2 compares the pollutant removal efficiency of CNT-based, MOF-based, and electro-catalytic membranes under various contaminant conditions.

Just like conventional membranes, MOF-based membranes are also prone to fouling. MOFs with highly porous surfaces even adsorb more unwanted background organics, leading to pore blockage and decreased performance over time. To improve antifouling, several efforts have been undertaken, such as embedding hydrophilic or zwitterionic polymers with MOFs, photocatalytic MOFs to degrade foulants under UV/visible light, and developing self-cleaning surfaces via advanced coatings [60,150,151,152]. Despite these promising lab-scale efforts, long-term field studies on MOF-based membrane fouling and degradation are still scarce. Overall, pilot-scale studies under realistic flow, pH, and actual complex contaminant loading are necessary to accurately evaluate the durability of the membranes and the maintenance requirements. MOF membranes used for adsorption or catalysis must be regenerated to remain economically and environmentally sustainable. However, regeneration methods such as acid/base washes, solvents, or thermal treatments can reduce the pollutant removal efficiency over time [153,154]. Moreover, MOFs that degrade or leach metal ions can pose secondary contamination risks [155,156]. Comprehensive ecotoxicological studies are needed to evaluate and mitigate this risk.

This not only undermines the overall stability of the matrix but also raises toxicity and environmental safety concerns [157]. Even though the creation of self-assembled networks through hydrogen bonding can minimize the nanofiller release, nanomaterial leakage still remains a critical concern. The extent of leakage is greatly dependent on the strength of the bond between the membrane polymer and nanoparticles. As these leachates or secondary pollutants are not well anchored, they pose health risks to both aquatic life and water consumers [158,159]. This way the nanoparticles can migrate from their deposition site to distant organs like blood, respiratory tracts, the gastrointestinal system, the brain, skin, and the liver, from where they may cause severe damage, including to the DNA [160,161].

Implementing MOF-based membrane technology to replace or complement conventional membranes is another critical challenge. Although MOFs can be synthesized at laboratory scale with well-controlled properties, their commercial-scale production remains expensive due to many factors, including the use of organic solvents (DMF, DEF, or ethanol), high temperature, and lengthy synthesis procedures. Moreover, incorporating MOFs into membranes introduces additional manufacturing steps (e.g., post-synthetic modification, particle dispersion, substrate pretreatment), raising production costs [153,162,163].

Among these challenges, water stability remains the most critical barrier, since structural degradation and metal leaching directly compromise both performance and environmental safety [164]. Scalability and MOF fabrication cost are the next major obstacles, as current synthesis routes for MOFs and their integration into membranes are still not economically competitive with conventional polymeric or ceramic membranes [89]. Interfacial compatibility and fouling are also important but can be partially mitigated through surface modification, polymer blending, or functional coatings [34]. Additionally, issues such as regeneration protocols and operational durability are secondary but still relevant, as they determine economic feasibility over multiple cycles.

In summary, MOF-based membranes offer immense potential for water purification, leveraging their tunable surface properties, high selectivity, and multifunctional capabilities. However, a number of significant challenges particularly stability, interfacial compatibility, scalable production, and durability under real-world conditions, must be overcome to move forward beyond laboratory demonstrations to industrial application. Overall, progress in this field hinges on (1) designing water-stable, tailed MOFs for selective removal; (2) economically scalable fabrication method development; (3) comprehensive life cycle assessment; and (4) pilot-scale testing under realistic operational scenarios.

## 4. Carbon Nanotube (CNT)-Based Membranes

**Figure 11:**
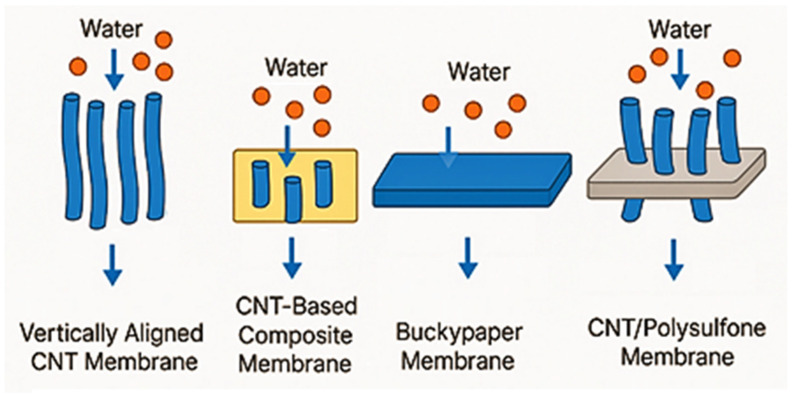
Schematic representation of CNTs-based membranes in different forms.

Since their discovery in 1991, CNTs have gained attention for their impressive mechanical and electrical properties, making them valuable for various applications, from energy to structural materials at micro and nano scales [165]. Micropollutants such as pharmaceuticals and personal care products are among the primary causes of water contamination. These compounds often persist in the environment even after going through multiple traditional water treatment methods. CNT-based membranes are those advanced materials that combine the physicochemical properties of CNT with the benefits of membrane technology. Through physical and/or chemical modifications, CNTs gain improved capacity of adsorption or rejection rate, selectivity toward targeted pollutants, higher flux, etc. As illustrated in Figure 11, this type of membrane includes vertically aligned CNT membranes (VACNT), buckypaper membranes, and CNT-based/supported composite membranes, among a few others that have shown great promise in working as adsorbents for organic pollutants, in thte removal of dyes, heavy metals, oils, MPs, and organic pollutants, and in the desalination process [64,166,167,168]. However, potential environmental and health risks associated with the unintended release of CNTs from membranes remain an important concern. For instance, Alfei et al. reviewed that CNTs released into aquatic environments can accumulate in organisms and exhibit toxicity depending on their surface modifications [169]. Similarly, studies on CNT-metal hybrids show significant toxicity to algae when CNT hybrids are exposed in water [170]. Addressing these issues through leakage testing and ecotoxicological evaluation is essential to ensure the sustainable and safe application of CNT-based membranes. In addition, thin-film composite (TFC) and thin-film nanocomposite (TFN) membranes have gained considerable attention in recent years. As stable performance and high selectivity can be maintained using TFC membranes, they are often used in nanofiltration and RO [157].

### 4.1. Properties of CNTs for Membrane Application

CNTs are tiny cylindrical structures made from carbon atoms arranged in a hexagonal lattice. They primarily exist in two forms: single-walled carbon nanotubes (SWCNTs) and multi-walled carbon nanotubes (MWCNTs). Due to their exceptional electrical conductivity, thermal stability, high mechanical strength, low density, and tunable surface resistivity, CNTs are highly suitable for a wide range of filtration and separation processes. Their properties make them especially valuable in applications requiring electroactive or thermally tunable membrane systems [171,172]. The addition of CNTs with porous graphene membranes has shown extraordinary results, primarily due to CNTs having the ability to permit smoother water flow and better purification; nonetheless, the CNTs must be electrochemically activated for this. Electrochemically active CNT membranes are highly effective at adsorbing chemical and biological contaminants, mainly through electro-oxidation of the adsorbed contaminants. They also perform well in ion separation, thanks to their great flexibility and larger surface area. This allows the CNT-based membranes to outperform conventional polymer membranes by nearly 3 times. Additionally, with narrow pores, the CNT membranes can even separate the (Na^+^/Cl^−^) ions, positioning them as a highly suitable medium for desalination. Despite their high effectiveness, their performance depends heavily on the careful design and optimization of both the CNTs inside the membrane and the overall filtration process itself [173].

Adding metallic oxides like cobalt, iron, and cobalt ferrite to the MWCNTs, a composite can be made, and based on the concentration of these metallic oxides, the absorption capacity varies. Adjusting the concentration of CNTs and thickness of the material, the absorption efficiency can be greatly improved [174,175]. CNTs are generally placed in membranes either in raw form or in oxidized form. However, it is to be noted that during preparation of the CNT-enriched membrane, oxidized CNT membranes experience greater leakage of CNTs compared to raw CNT membranes. This may cause a lower thermal stability and mechanical strength for the oxidized CNT membranes compared to both the pure polysulfone (PSF) membrane and raw CNT membrane. Other than porosity and surface hydrophilicity, thermal stability, stress, and strain also depend upon the CNT content present inside the membrane. Interestingly, for the same amount of CNTs, the overall porosities can be different, as a study showed that the raw CNT membrane can reach a 54% porosity while the oxidized CNT membrane has 68% porosity [176]. Some key properties of different types of CNTs, which directly influence their performance when incorporated into various membranes in water purification systems, are shown in Table 3.

### 4.2. Fabrication Approaches and Structural Variation

**Figure 12:**
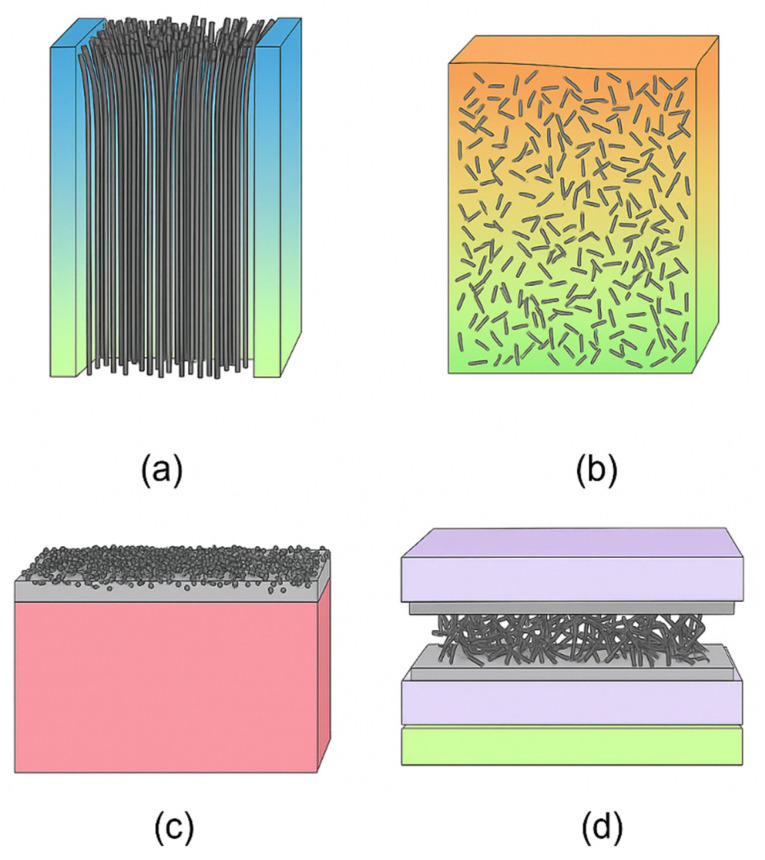
Structural variations in CNT-based membranes: (**a**) vertically aligned CNT membrane, (**b**) mixed-matrix CNT membrane, (**c**) CNTs are coated on membrane surface or support, and (**d**) CNTs are coated on membrane surface (support) as an intermediate layer.

Although CNTs are superior compared to traditional methods, their industrial adoption remains limited. This is primarily because of the high production cost and complexity of the fabrication process. Common methods for CNT production are chemical vapor deposition (CVD), arc discharge, and laser ablation. Also, techniques like high-pressure carbon monoxide (HiPCO), plasma-enhanced chemical vapor deposition (PECVD), electrolysis of molten salts, hydrothermal synthesis, etc., are often adopted for CNT synthesis. Among these fabrication processes, CVD allows for higher-quality productions that show better separation and purification abilities [177], especially for the purification of fertilizers, pesticides, industrial wastes, domestic sewage, etc. [2,3]. The fabrication of electroconductive membranes from CNTs is generally performed by the dry spinning method. In this method, CNTs are layered over a VACNT forest, forming a dense and conductive membrane structure, as shown in Figure 12a. To optimize the membrane’s performance for water purification, their pore size (about 28 nm) and membrane selectivity are tuned by controlling the dry layering and orientation of the CNT sheets. By implementing this type of membrane with electrochemical and ultrafiltration processes, water purification permeance could reach up to a value of 2.77 × 10^3^ L·m^−2^·h^−1^·bar^−1^ while maintaining an electrolytic constant up to 46.5 × 10^−3^ min^−1^ [178]. This result is 1.4 to 39 times larger than previously recorded values.

By using a combination of AC and DC electric fields, CNTs can be aligned and concentrated in a solution, resulting in membranes with a much higher density of CNTs. These allow the VACNT density to be two orders of magnitude higher than the density achieved from direct suspension of CNTs in liquid oligomer. Plasma etching is further performed to make the VACNT more permeable and have consistent pore sizes [179]. In parallel, innovations in membrane evaporator design have shown great results. For instance, graphene oxide-based membrane evaporators, fabricated using a spray-assisted technique, demonstrated efficient solar-driven water purification with an evaporation rate of 2.1 kg·m^−2^·h^−1^ and a conversion efficiency of 90.1% under one sun irradiation. This evaporation rate had been simulated under seawater, dye-contaminated water, lake water, etc., with the evaporation rate reaching as high as 8.2 kg·m^−2^ with salinity being below the WHO standard of 1.2 mgL^−1^ Na^+^ ions [180].

Introducing CNTs in fiber-enriched polymer composites, much stronger and more durable membranes have now been developed, as shown in Figure 12b. The molecular-level interactions between CNTs and the fiber matrix significantly enhance the key properties such as mechanical strength, stiffness, and electrical conductivity. Swelling is also another challenge occasionally seen in membrane systems, as it compromises the structural stability. To address this issue, membranes have been developed by mixing MXene and CNTs through a thermal cross-linking process, which bonds the materials together in a stable structure while leaving tiny channels for water to pass through [181]. By maintaining the optimal density of CNTs inside the membrane, the ultra-fast water flux can be ensured. If low density of CNT is given, then water flow is actually retarded [182].

Another innovative and cost-effective CNT-based membrane created with keeping the CNT’s scalability issues in mind is the carbon nanotube-adsorptive dynamic membrane (CNT-ADM), as presented schematically in Figure 12c,d. When applied as a surface (top) layer, carbon nanotubes (CNTs) enhance membrane performance by acting as a protective and functional filtration barrier. The CNT layer can improve resistance to fouling due to its tunable surface chemistry and hydrophobic or functionalized nature. And, while the CNT layer is used as an intermediate layer within membrane structures, CNTs serve critical roles in enhancing interlayer bonding and overall membrane stability. The presence of a CNT layer between active and support layers creates nanochannel pathways that enable faster water transport and lower resistance to flow. This configuration can help modulate pore size distribution and improve the trade-off between permeability and selectivity.

These types of membranes are fabricated by the application of the forward filtration process on the ultrafiltration membranes. For proper evaluation of the preparation of this membrane, factors such as CNT loading amount, preparation time, and transmembrane pressure (TMP) were noted. An agitation speed of 50 rpm along with 1 bar TMP and a CNT loading amount of 55 g/m^2^ with 10 min preparation time resulted in the CNT-ADM with the best optimizations. Unlike conventional membranes, pollutant removal is mainly performed through adsorption, which minimizes the direct contact between the membrane surface and the micropollutants. This results in less sedimentation on the membrane surface, meaning overall less fouling in the system, and extends the membrane’s operational lifespan. Additionally, when the CNT layers become saturated, regeneration of a new CNT layer can be performed through backwash cleaning. This not only restores the membrane’s adsorptive capacity but also repairs any of the structural leakages that may have occurred [183]. To ensure proper functioning of the CNTs incorporated with membranes, in many cases, the CNTs need to be either functionalized or transformed into different forms, depending on their fabrication processes. Table 4 summarizes the fabrication method-dependent differences in CNT properties toward their integration with the membranes for advanced water purification [184].

**Figure 13:**
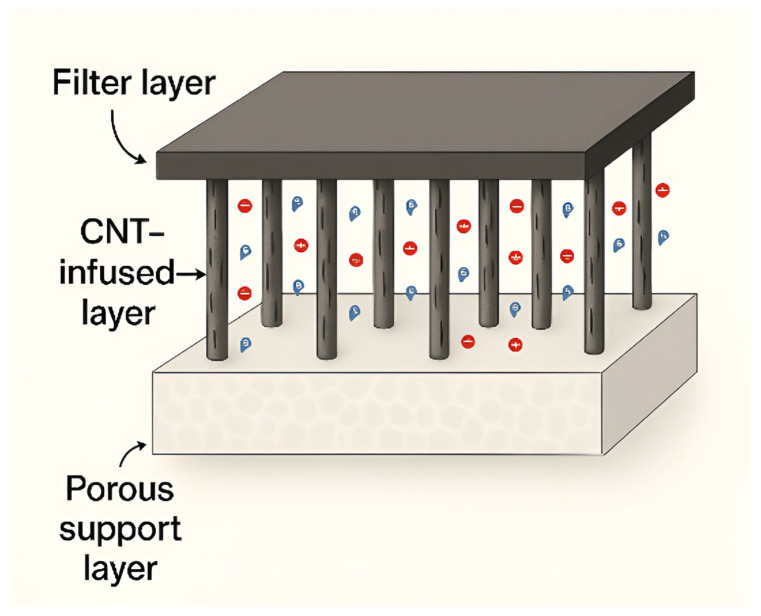
Schematic of triple-layer nanocomposite membrane.

To achieve a higher economic benefit without compromising its performance, a polymer called polyethersulfone (PES) is being used widely in CNT-based membrane fabrication. As shown in Figure 13, CNTs are incorporated into a PES matrix by a membrane distillation (MD) process, a sandwiched structure is formed by placing a CNT layer in between two layers of PES filters by an electrospinning method. Such triple-layered membranes have high porosity and surface hydrophobicity [185]. For the separation of organic solvents, the thin-film composite (TFC) membrane uses polytetrafluoroethylene (PTFE) as a base. In a similar fashion, to enhance the performance, a layer of CNTs is added between the PTFE and polyamide (PA) layer. This results in a strong and resistant barrier that is highly efficient in separating or rejecting unwanted substances. These new thin film composite organic solvent nanofiltration (TFC OSN) membranes showed higher dye rejection (the rejection of Bright blue B > 97%) compared to the previously developed TFC membranes, along with a stable methanol permeated flux of approximately 8.0 L·m^−2^·h^−1^·bar^−1^ [186]. It is worth mentioning that a new radiation-induced “grafting to” technique is now used to attach polyvinyl alcohol (PVA) to MWCNTs using gamma-ray irradiation at room temperature. With the assistance of a vacuum, the CNT-based composite membranes have been developed by depositing the MWNTs on cellulose acetate (CA) microporous membrane [187]. This modification resulted in a high water-flux recovery (>86.5%) and almost constant filtration flux, thus improving the MWCNTs’ ability to mix in water and their stability.

### 4.3. Applications in Pollutant Removal

CNT-based membranes are also quite useful in solar desalination. By treating the surface of the CNT membranes under different conditions, the amount of evaporation performed by the membrane can be controlled. If CNTs are made hydrophilic, it disrupts the hydrogen bonds between the water molecules and increases the evaporation rate. A study reported the optimal thickness of the membrane to be 1.97 nm for water evaporation [188]. CNT mixed membranes are also able to conduct electricity; hence, these can facilitate electrochemical reactions sometimes required to break down the otherwise stable compounds of the contaminants. These membranes work as anodes and have reportedly shown their effectiveness in breaking down organic pollutants in water. Due to micro-voids and CNTs, the permeability of these membranes can reach from 6.4 to 29.7 L/m^2^·h·bar. A modified membrane displayed an increased presence of -COOH, which enhanced its hydrophilicity, confirmed by FT-IR and XPS analysis [178,189]. Additionally, their zeta potential analysis proved the existence of a weaker negative charge compared to the unmodified membrane in a range of pH = 5–9.

Fouling is one of the major challenges that hamper the longevity of CNT membranes. Black liquor, a byproduct from the production process of paper, is one of the main contributors to fouling. Mixed matrix membranes (MMMs) are effective in reducing fouling. When MMMs are produced by mixing CNTs with polysulfone (PSF), the water flux increases significantly. MMMs containing 0.5% CNT performed best in separating lignin from black liquor, with a high rejection rate while maintaining good water flow [190]. Photo-regenerable CNTs have excellent self-cleaning properties utilizing a facile vacuum filtration method, followed by an FeOOH in situ anchoring and a silver amino reaction. These types of CNTs work better in the absorption of dyes (especially cationic dyes) compared to basic CNT membranes [191]. To minimize corrosion and combat fouling, stainless steel (SS) can also be introduced into the membrane system. SS offers strength, flexibility, and resistance to both corrosion and fouling. When CNTs are deposited onto SS substrates using a special electric field technique, the resulting stainless-steel carbon nanotubes (SS-CNTs) exhibit improved conductivity, enhanced antifouling properties, and better filtration performance [192]. However, a cautious approach should be adopted for such applications, as a CNT content more than the optimal limit may hinder the overall performance of the membrane [193].

Nanotechnology is becoming increasingly important across various fields, particularly in environmental remediation, where CNTs are being used for their pollutant removal capabilities. One major concern is organic pollutants like estrogen, a hormone released from both humans and animals. Large amounts of this estrogen, if present in the environment, hamper the physiological balance of aquatic and terrestrial organisms. Similarly, inorganic pollutants like heavy metals affect the tissue system in the human body. To combat these threats, CNT-based technologies, including adsorption, membrane filtration, and hybrid catalytic systems, have emerged [184]. The adsorption performed by the membrane mainly includes surface diffusion, pore diffusion, and adsorption reaction. Hybrid systems such as CNTs/O_3_ and CNTs/Fenton-like systems have also been introduced to fight against such pollutants. But they are only able to do so with the generation of reactive oxygen species (ROS). Moreover, CNTs are also able to be reduced to high-valent metal species like Fe(VI)/Mn(VII), which form intermediate metal-oxo species and greatly accelerate the decay of organic pollutants [194].

### 4.4. Advantages and Current Challenges

Vertically aligned CNTs with smaller diameters have tiny pores (0.8 nm) that allow water molecules to pass through them quickly, up to 100,000 times faster than traditional methods. Pores being opened through the plasma etching process facilitate the water transport. When comparing 0.8 nm and 3 nm CNTs, it was found that the smaller ones showed a much higher hydrodynamic slip length, going up to 8.5 μm as the diameter decreased [195]. With a high mechanical strength and structural stability, CNTs are highly effective in absorbing pollutants better than traditional methods.

CNT-based membranes have unique structures and properties at the nanoscale, but these benefits do not always translate to larger and practical separation applications. Often, challenges are faced in the distribution/orientation of CNTs in MMCNT membranes. However, it has now been proven that if proper modification or functionalization of CNTs can be implemented properly, not only do they improve in contaminant removal but also make the reusability and recyclability of the membrane materials easier. Structural engineering and functionalization of the CNTs play a very significant role in the removal of a wide variety of otherwise tenacious contaminants. Various methods used to enhance CNTs are oxidation, alkali activation, magnetic modification, grafting of metal or metal oxide catalysts, combining with other carbon nanomaterials, or attaching special chemical groups. The removal process of pollutants also changes depending on the type of functionalization induced on the system. Organic pollutants are removed through hydrophobic reactions, π–π bonding, and micropore filling, while polar or inorganic pollutants are removed through chemical reactions on the surface of the CNTs [196].

Despite their remarkable development in the removal of heavy metals, the prevalent deployment of CNT-based membranes is faced with a number of obstacles. These include proper functionalization of the nanotubes, ensuring even distribution, and control over the amount of CNTs present inside the membrane. Other concerns include integrating the active layer with the support structure, optimizing the number of pores, and managing the costs of making these membranes. The strength of the interaction between CNTs and polymers inside the membranes dictates how well the functionalized CNT membrane performs in water treatment. For proper functioning, it is crucial that the CNTs are both evenly distributed and securely bonded to the membrane structure. However, the precise effect of pore size, porosity, and surface properties on the membrane’s filtration ability remains unknown. Further investigations are required in this sector [197].

Packing nanoparticles in CNT-based membranes also shows notable challenges. Due to the high pressure drop caused by the nanoparticles, they cannot be packed in a straight column, limiting uniform distribution. So, to enhance their effectiveness, nanoparticles often need to be embedded in, coated on the surface, or combined with nanofibers. As nanofibers have high porosity and surface area, they contribute to their high adsorption and filtration capacity. But in the case of embedded nanoparticles, a slight reduction in adsorption efficiency is often observed. Another challenge is the proper mathematical modeling. Nowadays, membrane adsorption (MA) operations generally involve adsorption isotherms and kinetics. More characterization factors are necessary for better characterization of MA membranes [198]. However, overuse of nanomaterials is now responsible for creating nanophobia. To overcome this nanophobia, proper handling and disposal of these materials are required. In the case of CNTs, the extent of their toxicity and environmental hazards is still not properly known. So, it is urgent that a guideline be set up for the proper use of CNTs and that proper risk management measurements are taken [199].

Additionally, CNT materials themselves could also pose health risks due to CNT leakage. For this, CNT membranes, which are able to work long term under high pressure, need to be developed. Both the manufacturing processes and the manufacturing cost still remain as critical barriers that are stopping CNTs from being used in both small-scale applications and large-scale industrial use. Addressing these factors is a must to enable the practical deployment of CNT membrane technologies [200,201].

The CNT-based membranes show impressive results when it comes to rejecting dyes and pollutants. But the structure and performance of these membranes can still be further improved. The addition of MWCNTs to reduced graphene oxide (rGO) results in a membrane that allows water to pass through 4.8 times faster than regular rGO membranes [202]. Overall, these findings highlight that while CNT-based MMMs offer significant potential, there remains substantial room for optimization in terms of performance, scalability, and stability.

## 5. Electro-Fenton and Electro–Catalytic Hybrid Membranes

In general terms, electro-Fenton (EF) and electro–catalytic (EC) hybrid membranes represent an emerging class of advanced materials designed for efficient wastewater treatment and mitigation of harmful impacts on the environment. Since the EF process was first proposed in 1996, it has gradually emerged as an effective way of removing organic pollutants from water by breaking them down to small particles. The electro-Fenton process integrates electrochemically generated hydrogen peroxide with catalytic iron species to produce reactive hydroxyl radicals that cause rapid degradation of organic pollutants. Hybrid membranes combine this electro-Fenton mechanism with catalytic functionalities such as embedded nanoparticles, conductive polymers, or metal oxides within membrane structures. These membranes serve dual roles: acting as selective filtration barriers and active catalytic sites for pollutant degradation under an applied electric field. The synergy between electrochemical reactions and membrane separation enhances treatment efficiency, minimizes secondary pollution, and enables continuous operation in flow systems.

### 5.1. Concept of Reactive Electrochemical Membranes

**Figure 14:**
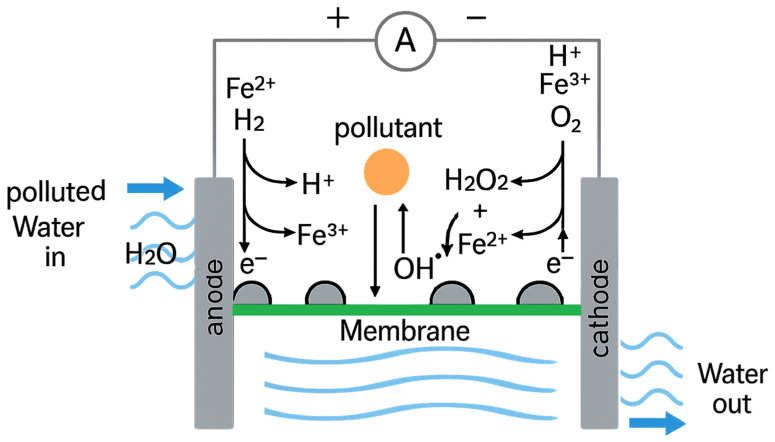
Schematic representation of a typical electro-Fenton membrane mechanism.

Electrolytic membranes (EMs) are a comparatively newer water treatment platform. It integrates membrane-based separation with electrochemical technologies in order to remove harmful pollutants from water [203]. Advanced oxidation processes (AOPs), like Fenton’s reagent, are effective methods for removal of biorefractory organic pollutants from wastewater by generating highly reactive oxygen species. In Figure 14, a generalized schematic of an electro-Fenton-based water filtration membrane is presented. In the electro-Fenton membrane system, water containing pollutants typically enters from the anode side. At the anode, oxidation occurs, releasing protons (H^+^) and electrons (e^−^), and may also produce Fe^3+^ ions when iron electrodes are used. This initiates the electrochemical environment necessary for the Fenton reaction. On the other side, at the cathode, oxygen is reduced, forming H_2_O_2_ in acidic conditions. This H_2_O_2_ diffuses towards the membrane, promoting continuous hydroxyl radical generation. Additionally, Fe^3+^ may be reduced back to Fe^2+^, sustaining the catalytic cycle. The membrane serves two roles: it filters pollutants and acts as a platform for in situ Fenton reactions. Here, Fe^2+^ reacts with hydrogen peroxide (H_2_O_2_) generated at the cathode to produce hydroxyl radicals (•OH), highly active oxidants that degrade organic pollutants from water.

However, some downsides of using Fenton’s reagent include the formation of iron sludge, high consumption of H_2_O_2_, and the requirement of precise pH control. To improve on these issues, electro-Fenton and heterogenous electro-Fenton (HEF) have been developed. HEF uses solid mineral catalysts that are efficient, stable, and environmentally friendly. However, the preparation of HEF is challenging due to the agglomeration of unsupported nanoparticles and complex preparation techniques [204]. To overcome this, researchers have developed a new iron- and nitrogen-doped carbon catalyst (Fe-N-C) with hemin, a pharmaceutical substance, as the source of carbon and KHCO_3_-MgO as a dual-porogen, a pore-forming agent. This dual porogen simultaneously improves porosity and exposes more active iron sites. While testing this system, it was observed that the system achieved a 93.82% degradation of ciprofloxacin (fluoroquinolone antibiotic substances) in just 50 min and 87.87% mineralization within 90 min [205].

Another HEF was developed using Fe^0^–Fe_3_O_4_ nanoparticles and cerium dioxide (CeO_2_) hollow spheres embedded in porous carbon made from skimmed cotton. Apart from having a hollow tubular structure due to the cotton-based carbon, it also had evenly sized hollow spheres on the surface due to having CeO_2_ spread across the system. A total degradation of 95.59% sodium was recorded in just 120 min, along with the removal of 95.21% Chemical Oxygen Demand (COD) in 240 min. The presence of zero-valence iron helps to speed up the catalyst’s activity and minimize secondary pollution [206]. Removal of antibiotics, especially enrofloxacin, from water needs an advanced type of reactive electrochemical ceramic membrane (RECM). This layer combines ceramic structure with a functional layer made from reduced titanium dioxide nanotubes and lead dioxide. Thanks to this design, high effectiveness and stability are noticed in the breakdown of enrofloxacin. The key element in the degradation of these antibiotics is the generation of reactive oxygen species [207].

**Figure 15:**
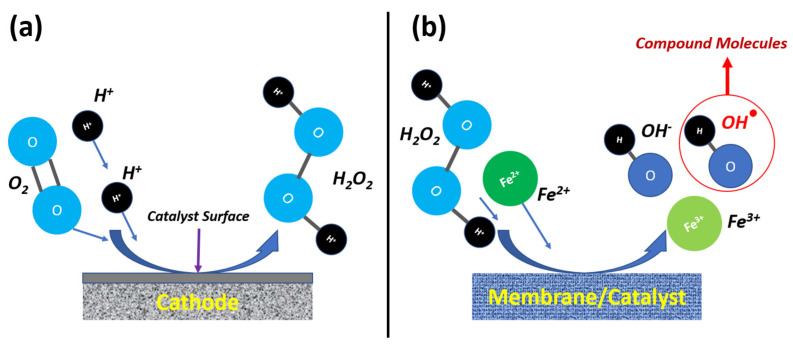
Schematics of (**a**) reduction of oxygen molecules (O_2_) into hydrogen peroxide (H_2_O_2_) on the cathode and (**b**) production of hydroxyl radical (OH•) through Fenton reaction in the presence of Fe^2+^ ions on the membrane with catalytic properties.

The electro-Fenton process mainly relies on two different types of catalysts: ones on the cathode surface that facilitate the reduction of oxygen into H_2_O_2_, via 2-electron transfer oxygen reduction reaction (ORR) mechanisms, as in Figure 15a. Another type of catalysts on the membranes that support Fenton-type catalyst for generating OH• radicals from H_2_O_2_, as shown in Figure 15b. The search for materials that can partake in both these processes has led to the development of bifunctional electrocatalysts (a mixture of carbon microspheres with Eco-graphene). This bifunctional catalyst is able to degrade various pollutants, including tetracycline (TTC), and achieves up to 83% degradation [208,209,210]. For further degradation, a microbial fuel cell (MFC) can be attached and used along with the EF process. Using an air cathode created by mixing activated carbon/graphite powder with PVDF binder resulted in higher H_2_O_2_ generation with slower Fe^3+^ reduction compared to the MFC carbon felt cathode. In the case of MFC-coupled EF, the Rhodamine B removal rate constant and mineralization current efficiency were found to be increased by 64% and 42%, compared to the standalone EF system. This hybrid system also helps in power density, due to the higher Fe^3+^/Fe^2+^ redox potential [211].

### 5.2. Design of Conductive Membranes

The efficiency of EF and EC processes greatly depends on the electrical conductivity of the membranes. It is crucial for these membranes to have excellent electron mobility while also promoting chemical reactions. To speed up the chemical reactions, a membrane called an electrocatalytic composite membrane with a deep permeation nanostructure (DPNS) has been created. The boosted electron transfer in this membrane is due to the embedded MnO_2_ particles [212]. Among the different structures of EF membranes, the membranes with honeycomb-shaped structures exhibited a higher efficiency. The special EF membrane created by growing a catalyst called FeNi layered double hydroxide (FeNi LDH) on a CNT membrane improves the ability to produce OH• radicals [213].

The hydrophobicity of the cathode and generation of H_2_O_2_ are greatly influenced by the shape of the cathode used in the system. An experimental approach Liu et al. used graphite powder, boric acid, PTFE, and an adhesive agent to form a lotus-leaf-like super-hydrophobic surface and then molded it into a lotus-leaf-like shape using copper wire meshes [214]. Being excellently hydrophobic, this unique structure greatly enhanced the H_2_O_2_ generation, leading to an improved EF process. In performance text, wastewater treated using this material system presented a Chemical Oxygen Demand (COD) removal of 76.8% and TOC removal of 73.5%. Moreover, this process also has great potential to act as a pre-treatment step in enhancing the biodegradability of pollutants before biological treatment.

Another method called Flow-Through Electrochemical Membrane Filtration (FEMF) has also provided great results in the removal of persistent organic pollutants (POPs) while also maintaining minimal fouling. The combination of electrochemical oxidation (EO) and membrane filtration makes this possible. The reactive electrochemical membrane (REM) anode is made using an activated carbon fiber belt (ACFF) with a PVDF membrane. Furthermore, due to ACFF having high absorptive capacity, pollutants like aniline are pulled into the active region, where they are then broken down. With low energy use of 1.51 kWh/kg across a cell voltage of 3.0 V, the system was able to achieve 97.5% pollutant removal. After adsorption, the pollutants are then decomposed using direct electron transfer (DET) and ROS, which allows the membrane to regenerate its adsorptive ability [215].

### 5.3. Different Aspects on EF-Based Pollutant Degradation Processes

On some occasions, a basic electro-Fenton process may not be enough to break down highly stable pollutant molecules like perfluorooctanic acid (PFOA). For PFOA, a method called solar photo-electro-Fenton (SPEF) has been designed. This method uses a special membrane made from MOFs and nanofibers by the electrospinning process. This helps to generate H_2_O_2_ and OH• radicals that can degrade PFOA efficiently. What makes this approach more promising and reliable than others is the fact that a 99% degradation can be achieved in 120 min [216]. Even though 99% degradation can be achieved, a good amount of time is required. To speed up this process, a combination of ultrasound and fluorinated ethylene propylene (FEP) can be used. This generates H_2_O_2_ much faster than traditional methods, and the addition of iron (Fe III) enhances the chemical reactions [217]. Another catalyst made from carbon materials doped with cobalt and nitrogen shows amazing results in the removal of tetracycline hydrochloride (TC) and also generates sufficient H_2_O_2_ and breaks it down to OH• radicals. This catalyst, being made mostly from carbon, does not release any harmful metals during the treatment process [218].

For removals of nanoscale antibiotics, nanoarray-structured EFM (NS-EFM) is often considered. Nanowires are typically used in the fabrication process of this membrane for better chemical reactions and efficient movement of the radicals. This membrane has the ability to remove the antibiotic sulfamethoxazole (SMX) by 99.4% with low energy use [219]. In the field of desalination of water, MD is a relatively newer approach. It has shown strong potential in filtering a variety of contaminants. However, one of its biggest limitations is that it struggles to completely remove volatile organic compounds (VOCs) from the water. VOCs can sometimes permeate through the membrane along with water. To address this, the integration of the EF process with MD has proven to be quite beneficial. The EF process causes a barrier to be built on the surface of the membrane, which entraps the VOCs and helps in their degradation while allowing water particles to pass through [220].

Breaking down organic dyes in industrial wastewater can be completed conveniently in the EF process. Along with that, some membranes also have self-cleaning abilities. This not only reduces the fouling but also allows water to pass through easily without letting any oils or dyes pass through [221]. Superior pollutant removal can be achieved by the mutual integration of EF and EC in a hybrid configuration. In this process, the product from the EC process is fed to the EF process, allowing for sequential degradation. In a study by Mirzaei et al., the hybrid system achieved a Chemical Oxygen Demand (COD) removal of 55% and Total Suspended Solids (TSS) removal of 96% during the EC stage, followed by an additional removal of COD by 66.2% in the EF stage. Interestingly, this hybrid system not only improved overall performance but also lessened the consumption of power compared to operating these two processes separately. This makes this hybrid system a much more energy-efficient solution for water treatment [222].

An interesting way to clean wastewater that can simultaneously produce electricity at the same time is the bio–electro–Fenton (BEF) system. By combining the microbes at the anode with the electrochemical Fenton reactions at the cathode, this system works to break down harmful pollutants like POP and other contaminants. Other major factors for using this system are the auto-generation of H_2_O_2_ and production of power during the treatment process [223].

### 5.4. Advantages and Current Challenges

Electro-Fenton and electro–catalytic-based membranes are a smart and efficient solution for wastewater treatment by performing both the physical filtration and the chemical breakdown of pollutants. This dual action not only minimizes common issues like membrane clogging but also reduces maintenance needs and increases operational lifespan. Upon integration with advanced oxidation processes (AOPs), their effectiveness is significantly enhanced, making them a strong candidate for tackling POPs [224]. Another advantage of advanced electrochemical treatment methods is their effectiveness in microbial disinfection. In the treatment of raw dairy wastewater, a two-step process is needed. Only electrocoagulation is not enough; it needs to be followed by an EF or PEF process. EC alone shows limited efficiency. But when paired with EF or PEF, this combined process is able to destroy bacteria like Escherichia coli and enterococci. Upon further addition of UVA light, enhanced generation of ROS is seen, which is mainly responsible for microbial degradation [225]. POPs, which are notoriously hard to remove, are responsible for leaving behind harmful residues. Using BEF properly degrades these POPs and reduces the need for various toxic chemicals. Additionally, the reactor designs for BEF are extremely flexible with high customizability, environmentally friendly and cost-effective [226].

One of the main problems faced during electro-Fenton and electro–catalytic processes is the poor stability of the electrodes involved. Sometimes, even in treated water, there may exist some traces of toxic metals or catalytic nanoparticles [204]. Metal leakage may become a common problem if it is not properly handled, which may actually contaminate the water. Energy recovery is also another big issue with this system. These electrodes need a good amount of electricity to function properly, even in lab-scale experiments, which may be an obstacle for the mass deployment of these systems [227]. Another important part of the EF processes is the type of catalysts used. The best catalysts for this process are noble materials. But these materials are not cheap and not readily available. To overcome this, new and cheaper catalysts have been introduced [228]. While these low-cost catalysts show promising efficiency in the degradation of pollutants, they cannot yet match the performance illustrated by noble materials. As a result, there still exists a demand for such a cost-effective and high-performance catalyst that can reliably be used as an alternative to noble materials.

## 6. Critical Assessments on AFMs-Based Water Treatment

**Figure 16:**
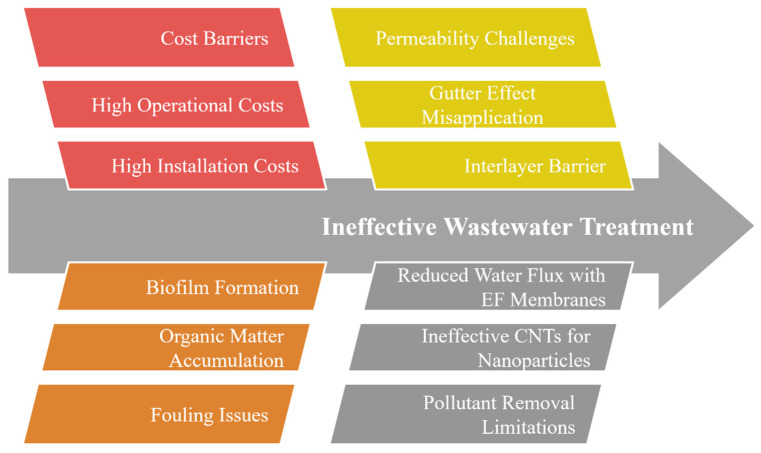
Challenges in membrane filtration for wastewater treatment.

The improper disposal of wastewater from industrial, agricultural, and domestic sources has become a major environmental concern, as it often contains toxic heavy metals and harmful emerging contaminants. These contaminants are resistant to natural degradation, making effective wastewater treatment essential to ensure that discharged water complies with environmental standards. Among available methods, membrane filtration offers a simple, reliable, and rapid solution for removing a broad spectrum of contaminants. However, the adoption of AFMs still faces several unresolved challenges (Figure 16). High installation and operational costs remain a significant barrier, particularly for small- and medium-sized enterprises [229]. A critical technical challenge is membrane fouling, caused by the accumulation of organic matter, biofilms, and other pollutants on the membrane surface. This not only reduces water flux and filtration efficiency but also leads to irreversible membrane damage over time. Even minor fouling can significantly impair performance and contaminant removal. Therefore, implementing regular maintenance and effective anti-fouling strategies is essential to ensure the long-term viability of AFMs in both industrial and general water treatment applications [230].

The use of active-passive membranes combining passive anti-fouling strategies with active self-cleaning mechanisms offers a promising direction for mitigating fouling-related challenges. In such systems, passive methods help reduce fouling accumulation, while active techniques effectively remove oil and other deposits from the membrane surface [231]. To further enhance membrane permeability, interlayered thin-film nanocomposites (TFNi) incorporating materials like carbon nanotubes (CNTs) or metal–organic frameworks (MOFs) have been developed. These interlayers facilitate the so-called “gutter effect,” which allows water to flow more rapidly through the membrane. However, if not carefully designed, the interlayer can inadvertently act as a barrier to water flow, hindering overall performance [232,233]. In the case of ultra-fine contaminants such as nanoparticles, CNT-based membranes may occasionally underperform. For such situations, electro-Fenton (EF) membranes provide an effective solution due to their high removal efficiency. Nonetheless, this comes with a trade-off in the form of reduced water flux. Interestingly, the integration of CNTs into EF membranes not only restores water permeability but also enhances pollutant rejection, offering a synergistic advantage [234].

Traditionally, TFC membranes are faced with challenges that result in a trade-off between permeability and selectivity. This limited their desalination of water purification performance. For this, TFNs had been used, which are specifically designed with functionalization to enhance water flux and improve selectivity [235]. These TFNs can be used under various conditions for thermal stability, chlorine resistance, antimicrobial activity, and dye removal [236]. By incorporating CNTs and MOFs in such TFN membranes, it is possible to further enhance the water treatment capabilities. The addition of carboxyl-functionalized multiwalled carbon nanotubes (COOH-MWCNTs) in the support layer of TFCs improved hydrophilicity, stability, and surface charge. This resulted in a 161% increase in water flux with almost 92% recovery [237,238]. The addition of CNTs also helps in the formation of defect-free top layers, resulting in a higher percentage of dye rejection with long operation stability compared to control TFC or TFN membranes [186,239].

Similarly, the addition of an MOF layer also has its benefits. MOF-based TFC MMMs can be fabricated using the 3D electrospray printing process. This enables the creation of defect-free membranes with 2–3 mm thickness and tunable pore size [240]. In addition, MOFs are also used as sacrificial interlayers during interfacial polymerization in TFN membranes. The MOF particles degrade as soon as they come in contact with water and create nanovoids to enhance water pathways [241]. Introduction of MOF also leads to strong rejection of small neutral contaminants and salts along with strong antifouling performances [242,243].

MOF-based membranes, despite their promising properties such as tunable porosity, high surface area, and selective permeability, face several key limitations in water filtration applications. One major challenge is their structural instability in aqueous environments, particularly under varying pH conditions, which can lead to degradation and reduced performance. Additionally, the presence of defects, poor interfacial compatibility with substrates, and difficulties in large-scale fabrication hinder their practical deployment. To address these issues, strategies such as post-synthetic modification to improve water stability, incorporation of MOFs into polymer or ceramic supports to enhance mechanical strength, and the development of defect-engineering techniques for better membrane integrity have been proposed. Moreover, adopting scalable fabrication methods like layer-by-layer assembly or interfacial synthesis may support their transition from laboratory to industrial use.

To overcome the diverse challenges associated with treating the wide range of pollutants present in wastewater, while also aiming for compactness and energy efficiency, current research is increasingly focused on integrating engineered nanomaterials (ENMs) into decentralized wastewater treatment systems [244]. Materials such as carbon nanotubes (CNTs), metal–organic frameworks (MOFs), and redox-enhanced membranes (REMs) are gaining attention due to their excellent catalytic activity, reusability, and adaptability under varied operating conditions. The goal extends beyond simply minimizing the use of harsh chemicals; it also involves addressing real-world issues such as scalability, cost-effectiveness, energy consumption, and accessibility for both rural and urban settings. Table 5 presents a comparative overview of CNT-based, MOF-based, and electro-Fenton/electro–catalytic hybrid membranes, detailing their respective strengths and limitations in the context of practical water treatment applications.

## 7. Environmental and Economic Considerations

**Figure 17:**
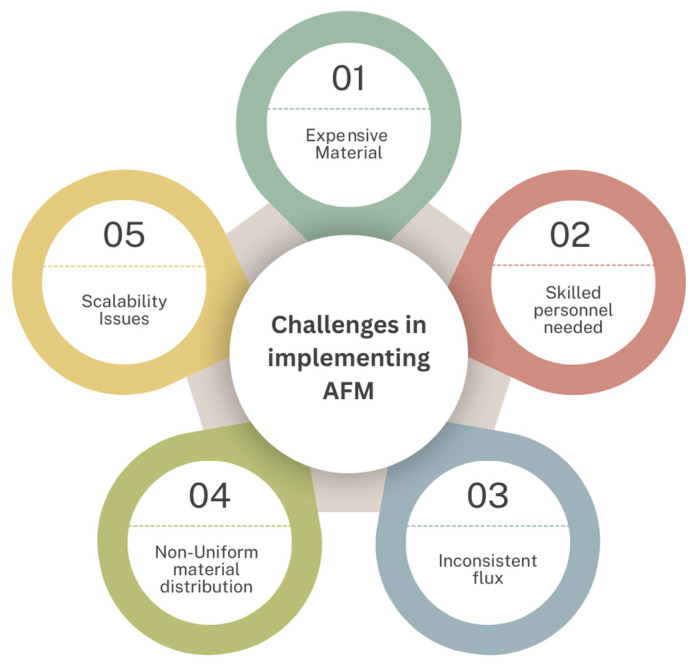
Challenges in implementing advanced membrane technologies.

Untreated water poses serious risks for both human health and the environment. Wastewater can originate from industries, agriculture, and urban runoff, often contaminating rivers, lakes, and groundwater, giving rise to an unhealthy and disrupted ecosystem [277]. Over the years, different ways of treating polluted water have been introduced. Traditional ways like sedimentation, coagulation, etc., are often time-consuming and energy-intensive with limited effectiveness. As a response to these problems faced in traditional processes, new membrane technologies are now being discussed. Laboratory studies involving AFMs used individually or in combination have demonstrated sound removal effectiveness over a wide range of pollutants. However, when brought to real-world on-site applications, their performance, which is still better than traditional methods, does not have the same accuracy as the in-lab results. Despite their huge potential, industries are still unsure about their implementation. Cost and scalability are the two huge barriers in this system, shown in Figure 17. The fabrication of AFMs involves advanced materials like CNTs, MOFs, or electro-catalytic components, which are expensive and require skilled personnel for maintenance and operation. On the other hand, when produced on a larger scale, the material is not always distributed uniformly, leading to inconsistent flux and pollutant removal zones across the membrane. Another critical drawback for AFMs is their limited lifespan. As the AFMs undergo degradation over time, they end up releasing nanomaterials, raising the toxicity of the treated water [278,279,280].

The use of electrocatalytic dual-membrane filtration (EDMF) offers a lot of significant environmental advantages. It allows for flexible control over degradation mechanisms to suit the different types of water to be treated. Direct and indirect oxidation processes are also regulated by using both the anodes and cathodes simultaneously. This reduces the potential environmental harm by a lot. Moreover, having the ability to optimize electrodes and membrane configuration reduces both energy consumption and material waste, making it a more green and suitable water treatment method [281].

CNT-based membranes are able to show high operational lifespans due to their robustness, high electrical conductivity, and comparatively larger surface area. This helps its repeated use in processes that require separation. These high operational capabilities can be further improved through chemical modifications [40,166]. Although these membranes show excellent performance, their lifecycle is not only limited to operational stability. Their fabrication, modification, end-of-life management, and recycling also play a part in their lifespan [282]. With high flux and strong rejection, MOF membranes are seen to retain their structural integrity even after 12 months of immersion [60]. Moreover, EF membranes work better under gentle conditions. So, a longer lifespan can be maintained as long as minimal stress is generated on the materials [283].

While the MMMs offer significant benefits, concerns remain regarding their potential toxicity. Studies show that CNTs may induce oxidative stress, inflammation, DNA damage, and fibrosis depending on the purity, type, and surface chemistry [284]. These chemical compositions, stability, and other physiochemical properties also greatly influence the toxicity of MOF membranes. Depending on the nature of organic linkers and metal clusters, the toxicity levels vary [285]. On the other hand, EF membranes generate toxic intermediates. However, these byproducts are later broken down as the reaction moves forward [286].

In summary, while AFMs and their hybrid systems offer impressive environmental benefits, they also introduce challenges related to cost and long-term sustainability. The addition of nanomaterials to the AFMs, although effective, comes with its own set of challenges regarding toxicity and disposal. Economically, these technologies are still limited to laboratory experiments. Moving forward, balancing performance with affordability and environmental safety is a crucial step towards making AFMs ready for widespread use. With continuous innovation in materials science, membrane designs, and supportive frameworks, AFMs have the potential to become an effective, practical, and accessible solution for sustainable wastewater treatment on a larger scale.

## 8. Conclusions

The rapid growth of the global population, coupled with accelerated industrialization, has significantly contributed to the generation of large volumes of wastewater. Much of this wastewater is difficult to treat due to the presence of persistent organic and inorganic pollutants. As a result, there is an urgent need for water treatment technologies that are both efficient and reliable. Traditional methods often fall short in addressing these complex contaminants, which has led to growing interest in Advanced Functional Membranes. AFMs are specifically engineered to not only filter a broad spectrum of pollutants but also degrade them chemically at the active sites embedded within the membrane structure. Their tunable surface characteristics allow for extensive customization, enabling them to perform effectively under a variety of environmental conditions. Furthermore, the integration of AFMs into hybrid treatment systems, where they are combined with other technologies, has demonstrated synergistic effects and can enhance overall performance compared to individual systems operating in isolation.

Despite their promising potential, AFMs face several critical limitations that hinder their widespread adoption. Key concerns include high production and maintenance costs, scalability challenges, potential environmental risks associated with nanomaterial release, and questions surrounding their long-term stability. In addition, a general lack of public awareness and understanding of nanomaterials continues to pose a significant barrier to broader acceptance. To fully realize the potential of AFMs in water treatment, it is essential to prioritize advancements in material design, cost reduction strategies, and measures that ensure environmental safety. With well-structured planning and continued innovation, AFMs have the potential to become a transformative technology that may offer a safe, efficient, and reliable solution for achieving universal access to clean water.

Future studies should concentrate on interface engineering to improve compatibility between nanofillers and polymer matrices, which helps lower leakage and defects, rather than just focusing on small performance improvements. In order to reduce production-related environmental effects, green synthesis techniques such as solvent-free, low-energy, and sustainable fabrication methods are crucial. Finally, the development of membranes that are able to respond to changes, modify on their own, or are able to clean themselves automatically could greatly improve their ability to work under varying water conditions. By combining these strategies with initiatives for life-cycle management and recycling of end-of-life modules, these membranes will move more quickly from lab prototypes to workable, environmentally friendly water treatment solutions.

## Figures and Tables

**Table 1:** Physicochemical features of commonly used MOFs.

MOF	Metal Node	Organic Linker	Surface Area (m^2^/g)	Pore Size	Water Stability	Targeted Pollutants	Reference
ZIF-8	Zn^2+^	2-methylimidazole	~1600	~3.4 Å	Moderate–poor; hydrolyzes over time at ambient conditions	VOCs, dyes, some metal ions	[90,91,92]
UiO-66	Zr^4+^	Terephthalic acid	~800–1000	~6–8 Å	Excellent aqueous stability (acid/base)	Heavy metals (Cr^6+^), antibiotics, dyes	[93,94,95]
MIL-101 (Cr)	Cr^3+^	Terephthalic acid	~2700	29–34 Å (cage diameters)	Good hydrothermal stability and durability	Dyes, pharmaceuticals, antibiotics	[96,97]

**Table 2:** Comparative performance of different AFMs in pollutant removal.

Pollutant Type	MOF-Based Membranes	CNT-Based Membranes	Electro-Fenton/Electro–Catalytic Membranes	References
Heavy Metals	NH_2_-MIL-53(Al)/PAN shows 95% Co^2+^ removal	Removal up to 90–96% Cr^6+^, 70–90% Pb depending on CNT loading	Less common; mainly organic degradation	[139,140]
Dyes	ZIF-8/PVDF composite membranes remove 83.6% of Reactive Black, 95.8% of Methylene Blue, and 94.2% of Rhodamine B	Promotes removal by 30% when introduced	Electro-Fenton shows 80% dye degradation	[141,142,143,144]
Pharmaceuticals	>90% removal for both positively and negatively charged pharmaceutical pollutants	Removal more than 70% pharmaceutical pollutants	>85% removal for the treatment of pharmaceuticals in water	[139,145,146]
Organic micropollutants	MOF composites can achieve up to 99% rejection of volatile organic compounds (VOCs)	Can remove up to 79% Bisphenol A (BPA) while also rejecting 80% of DOC in seawater	Electro-Fenton achieves 97.68% COD removal with 82.41% salt recovery	[147,148,149]

**Table 3:** Table for properties of different types of CNTs.

CNT Type	Structure & Morphology	Key Properties	Advantages in Membranes	Challenges/ Limitations
SWCNT (Single-Walled)	Single graphene sheet rolled into a tube (diameter ~0.7–2 nm)	High surface area, high conductivity, excellent selectivity	High adsorption capacity, ideal for sensing and separation at molecular level	Difficult to purify, high cost, dispersibility issues
MWCNT (Multi-Walled)	Multiple concentric CNT layers (diameter ~5–50 nm)	Mechanically strong, thermally stable, cost-effective	Good for composite membranes, high chemical stability	Lower surface area than SWCNTs, less flexible for tuning
VACNT (Vertically Aligned)	CNTs aligned perpendicular to membrane surface	High flux, low flow resistance, directional transport	Superior water permeability, highly organized pore structure	Complex and costly fabrication
Buckypaper	Randomly entangled CNT sheets pressed into thin films	Freestanding membrane, moderate conductivity, porous	Easy to fabricate, scalable, can serve as standalone membrane or layer	Lower selectivity, needs reinforcement for mechanical strength
CNT-Polymer Composites	CNTs dispersed in polymer matrix (e.g., PSf, PES, PVDF)	Tunable porosity, mechanical strength, antifouling properties	Versatile, improves polymer membrane properties (flux, fouling, rejection)	Requires functionalization for dispersion, cost scaling
Functionalized CNTs	CNTs modified with -OH, -COOH, NH_2_, etc.	Improved dispersion, hydrophilicity, specific binding sites	Tailored for removal of specific contaminants, better integration with polymers	Functionalization may damage structure or reduce conductivity

**Table 4:** Fabrication method-dependent properties variations in CNTs.

CNT Properties	CVD	Template- Assisted CVD	Polymer Blending	In Situ Polymerization	Layer-by-Layer (LbL) Assembly	Direct Coating
Compatible Membrane Material	Inorganic	Inorganic	Polymeric	Polymeric	Polymeric	Inorganic or Polymeric
Bonding Mechanism	In situ growth	In situ growth	Weak interactions (Van der Waals, H-bonding)	Covalent bonding	Electrostatic and Covalent	Surface adhesion/covalent
CNT Arrangement	Aligned or Random	Aligned	Random	Random or Aligned	Mostly Aligned	Random
Mechanical Stability	Excellent	Excellent	Good	Poor	Good	Good
Common Defects	Impurities	Impurities	Poor dispersion	Low durability	Pinholes	Delamination/peeling
Scalability	Limited to substrate size	Limited to template size	Easily scalable	Limited by processing	Scalable with control	Limited to substrate size
Pore Size Control	Precise	Precise	Moderate	Precise	Precise	Moderate
Ease of Fabrication	Moderate	Complicated	Simple	Complicated	Complex	Simple
Practical Use	Industrially promising	Moderate	Suitable for general use	Experimental stage	Lab-scale and advanced studies	Simple but effective

**Table 5:** Comparison between CNT-based, MOF-based and electro-Fenton/electro–catalytic hybrid membranes.

Parameter	CNT-Based Membranes	MOF-Based Membranes	Electro-Fenton/Electro–Catalytic Hybrid Membranes	References
Main Mechanism	Physical filtration + Adsorption	Molecular sieving + Adsorption	Electrochemical degradation + Filtration	[245,246,247]
Typical Applications	Oil-water separation, dye removal, heavy metals	Heavy metals, dyes, PFAS, antibiotics	Pharmaceuticals, endocrine disruptors, pesticides	[248,249,250]
Contaminant Types Removed	Moderate range: dyes, oils, some metals	Broad range: organics, metals, emerging pollutants	Very broad: persistent organics, complex molecules	[251,252,253]
Rejection Rate	Moderate (60–90% for many contaminants)	High (up to 99% for targeted pollutants)	Very high (>99% for organics)	[149,254,255]
Water Flux	Very High (found up to ~1000–5500 L·m^−2^·h^−1^·bar^−1^)	Moderate to high (~30–250 L·m^−2^·h^−1^·bar^−1^)	Variable (depends on electrochemical setup)	[250,256,257,258,259,260]
Energy Requirement	Low	Low to moderate	High (due to electrical input)	[255,261,262]
Fouling Resistance	Moderate (requires functionalization)	Moderate (susceptible to pore blockage)	High (self-cleaning due to oxidation)	[263,264,265]
Material Cost/Scalability	Moderate to High (due to CNT synthesis)	High (MOFs expensive, sensitive to water)	High (complex fabrication, power requirement)	[253,266,267]
Strengths	High permeability, tunable surface, light weight	High selectivity, customizable pore structures	Complete breakdown of pollutants, no secondary waste	[268,269,270]
Limitations	Costly synthesis, limited removal of micropollutants	Stability in aqueous media, scalability	High energy use, electrode degradation	[271,272,273]
Reusability	Good (if fouling is controlled)	Moderate (regeneration needed)	Excellent (can be self-regenerating)	[274,275,276]

## Data Availability

Not applicable.
